# Molecular basis and dual ligand regulation of tetrameric estrogen receptor α/14-3-3ζ protein complex

**DOI:** 10.1016/j.jbc.2023.104855

**Published:** 2023-05-22

**Authors:** Bente A. Somsen, Eline Sijbesma, Seppe Leysen, Karolina Honzejkova, Emira J. Visser, Peter J. Cossar, Tomáš Obšil, Luc Brunsveld, Christian Ottmann

**Affiliations:** 1 Department of Biomedical Engineering and Institute for Complex Molecular Systems, Laboratory of Chemical Biology, Eindhoven University of Technology, Eindhoven, The Netherlands; 2 Department of Physical and Macromolecular Chemistry, Faculty of Science, Charles University, Prague, Czech Republic

**Keywords:** Nuclear receptors, protein–protein interactions, Estrogen Receptor, 14-3-3 protein, PPI stabilization

## Abstract

Therapeutic strategies targeting nuclear receptors (NRs) beyond their endogenous ligand binding pocket have gained significant scientific interest driven by a need to circumvent problems associated with drug resistance and pharmacological profile. The hub protein 14-3-3 is an endogenous regulator of various NRs, providing a novel entry point for small molecule modulation of NR activity. Exemplified, 14-3-3 binding to the C-terminal F-domain of the estrogen receptor alpha (ERα), and small molecule stabilization of the ERα/14-3-3ζ protein complex by the natural product Fusicoccin A (FC-A), was demonstrated to downregulate ERα-mediated breast cancer proliferation. This presents a novel drug discovery approach to target ERα; however, structural and mechanistic insights into ERα/14-3-3 complex formation are lacking. Here, we provide an in-depth molecular understanding of the ERα/14-3-3ζ complex by isolating 14-3-3ζ in complex with an ERα protein construct comprising its ligand-binding domain (LBD) and phosphorylated F-domain. Bacterial co-expression and co-purification of the ERα/14-3-3ζ complex, followed by extensive biophysical and structural characterization, revealed a tetrameric complex between the ERα homodimer and the 14-3-3ζ homodimer. 14-3-3ζ binding to ERα, and ERα/14-3-3ζ complex stabilization by FC-A, appeared to be orthogonal to ERα endogenous agonist (E2) binding, E2-induced conformational changes, and cofactor recruitment. Similarly, the ERα antagonist 4-hydroxytamoxifen inhibited cofactor recruitment to the ERα LBD while ERα was bound to 14-3-3ζ. Furthermore, stabilization of the ERα/14-3-3ζ protein complex by FC-A was not influenced by the disease-associated and 4-hydroxytamoxifen resistant ERα-Y537S mutant. Together, these molecular and mechanistic insights provide direction for targeting ERα *via* the ERα/14-3-3 complex as an alternative drug discovery approach.

Nuclear receptors (NRs) are a family of structurally similar, mostly ligand-regulated, transcription factors that control a diverse set of biological processes, including metabolism, cell proliferation, development, and immunity (1, 2). Several small molecules, including steroid hormones, fatty acids, vitamin D, and thyroid hormones, regulate NR activity by binding the NR’s ligand binding domain (LBD) (3, 4). Given their central role in gene regulation, NRs are associated with a broad scope of diseases, including autoimmune diseases, metabolic disorders, and cancer (2, 5, 6). The involvement of NRs in various pathological processes, combined with their ability to bind “drug-like” small molecules, have made NR-targeting therapeutics extensively utilized in the clinic to treat diseases (4, 7). Most NR-targeting drugs antagonize the activity of the protein by orthosteric inhibition of native ligand binding to the LBD (4, 6). For example, 4-hydroxytamoxifen (4-OHT) inhibits ERα activity by displacing its natural ligand estradiol (E2, Fig. 1, *A* and *B*) (5, 6). Despite the huge successes of orthosteric NR inhibitors, there is a growing interest in targeting NRs in alternative manners (8, 9, 10, 11). Novel therapeutic approaches include ligands that target allosteric sites in the LBD (12, 13, 14), NR–DNA interactions (15, 16, 17), NR dimerization (18, 19, 20, 21), NR-cofactor interfaces (22, 23, 24, 25), or induce targeted NR degradation (26, 27, 28). Drugging NRs beyond the orthosteric site of the LBD offers entry points into selective NR targeting, fine-tuning of pharmacological effects, targeting of drug-resistant NRs, or addressing orphan NRs that lack an accessible ligand binding pocket (8, 9, 10).

**Figure 1:**
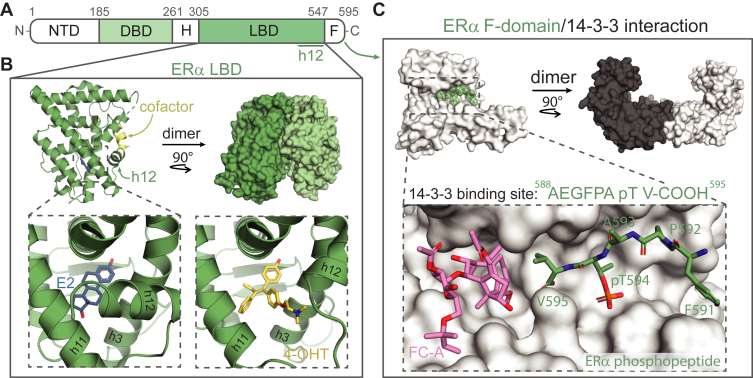
**The ERα/14-3-3 protein-protein interaction.***A*, schematic representation of ERα domains, including the N-terminal domain (NTD), DNA-binding domain (DBD), hinge-region (H), ligand-binding domain (LBD), and C-terminal F-domain (F). *B*, ERα LBD crystal structure (monomer, *green cartoon* and dimer, *green surface*). The binding of its endogenous ligand E2 (*blue sticks*) induces the folding of helix 12 (h12) in an active conformation, allowing cofactor binding (*yellow cartoon*). In contrast, antagonist 4-hydroxytamoxifen (4-OHT, *yellow sticks*) inhibits this conformational change and cofactor recruitment, PDB: 5WGD and 3ERT. *C*, crystal structure of 14-3-3 dimer (*gray*/*white surface*) and co-crystal structure of 14-3-3σ (*white surface*) with the ERα derived C-terminal phosphopeptide (*green sticks*) and small molecule stabilizer Fusicoccin-A (FC-A, *pink sticks*), PDB: 4JDD.

An alternative approach to modulate NR activity is *via* their protein–protein interaction (PPI) with the hub protein 14-3-3. These 14-3-3 proteins are known to interact, and thereby regulate, hundreds of different, mainly phosphorylated, protein partners. Within the large interactome of 14-3-3 are many well-known disease-related proteins such as the Raf kinases (cancer), LRRK2 (Parkinson’s), and CFTR (cystic fibrosis), which make 14-3-3 proteins highly involved in a larger variety of cellular processes and which makes 14-3-3 protein complexes highly interesting as drug discovery targets (29). Among the 14-3-3 protein partners are various NRs, including the steroid hormone-responsive Androgen Receptor (AR), Glucocorticoid Receptor (GR), and Estrogen Receptor alpha (ERα) (Figs. S1 and S2). Specifically, 14-3-3 binds AR upon phosphorylation of Ser213 in the N-terminal domain (NTD), thereby modulating AR transcriptional activity (30, 31, 32, 33, 34). Similarly, 14-3-3 binding enhances GR activity upon binding to pS134 in the NTD or pT524 in the LBD (35, 36, 37). In the case of ERα, 14-3-3 binds the phosphorylated penultimate residue T594 in the C-terminal F-domain (Fig. 1*C*), thereby reducing E2-dependent transcriptional activity (38). Cellular studies have further reported 14-3-3 binding to the DNA-binding domain (DBD) of ERRγ, the LBD of PPARγ, and ERβ, TRα, and PXR of which the exact binding sites remain unknown (39, 40, 41, 42, 43). These examples illustrate the diverse role that 14-3-3 plays in NR regulation and, by extension, the potential of targeting NR/14-3-3 PPIs.

Initial studies have shown that NR/14-3-3 PPIs can be modulated using small molecules. Specifically, the natural product FC-A or covalently tethered small molecules have been shown to stabilize the ERα/14-3-3 and ERRγ/14-3-3 PPIs (Figs. 1*C* and S3) (38, 44, 45, 46). These small molecules, also called molecular glues, increase the binding affinity between the two protein partners (PPI stabilization) by binding in a composite pocket formed at the interface of 14-3-3 and the phosphorylated NR protein. Further, stabilization of the ERα/14-3-3 PPI by FC-A suppresses ERα chromatin binding and subsequent transcriptional activity (38). These results illustrate the potential of the stabilization of the ERα/14-3-3 interaction as an alternative drug discovery entry.

Despite the potential of therapeutic targeting of NR/14-3-3 complexes, little is known about NR/14-3-3 interactions on a molecular level. To date, biochemical and structural studies of NR/14-3-3 complexes have been exclusively performed using short phosphopeptide mimics of the NRs (Figs. 1*C* and S2) (45, 46, 47). Studies of NR/14-3-3 complexes using protein domains or full-length NRs, such as those performed for other 14-3-3 PPIs (48, 49, 50, 51), are needed to gain an enhanced molecular and structural understanding of NR/14-3-3 complex formation. As an example, the use of the entire NR LBD allows studies on the interplay between NR/14-3-3 complex formation and aspects like NR dimerization, ligand binding, and cofactor recruitment. Additionally, small molecule stabilization of full-length NR/14-3-3 complexes can be investigated in the context of clinically relevant NR point mutations which is of high interest (52, 53, 54, 55). Molecular insights into NR/14-3-3 PPIs beyond the phospho-binding groove would also provide the potential to identify novel composite binding pockets for small molecule targeting, thereby expanding the number of entry points for novel PPI modulator design and increasing the potential for selectivity (56).

Within this work, we aim to enhance our understanding of 14-3-3 binding to ERα *via in vitro* characterization of the protein complex formed by 14-3-3ζ and an ERα construct comprising the LBD and phosphorylated F domain. To gain a robust understanding of protein complex formation and the stoichiometry of binding, several biophysical assays were performed, including analytical size exclusion chromatography (SEC) and analytical ultracentrifugation (AUC). In addition, differential scanning fluorimetry (DSF), fluorescence anisotropy (FA), and hydrogen-deuterium exchange (HDX) experiments determined the role of ERα ligands E2 and 4-OHT on ERα/14-3-3 complex formation and identified 14-3-3 binding to the drug-resistant ERα-Y537S mutant. Finally, we show that the ERα/14-3-3 PPI can be stabilized by the natural product FC-A and that this PPI stabilization can be achieved independently from ERα ligand binding in both wild-type ERα and the ERα-Y537S mutant, thus presenting a potential orthogonal therapeutic strategy for targeting endocrine resistance in breast cancer.

## Results

### Construct design and protein expression of ERα LBD-F domain

**Figure 2:**
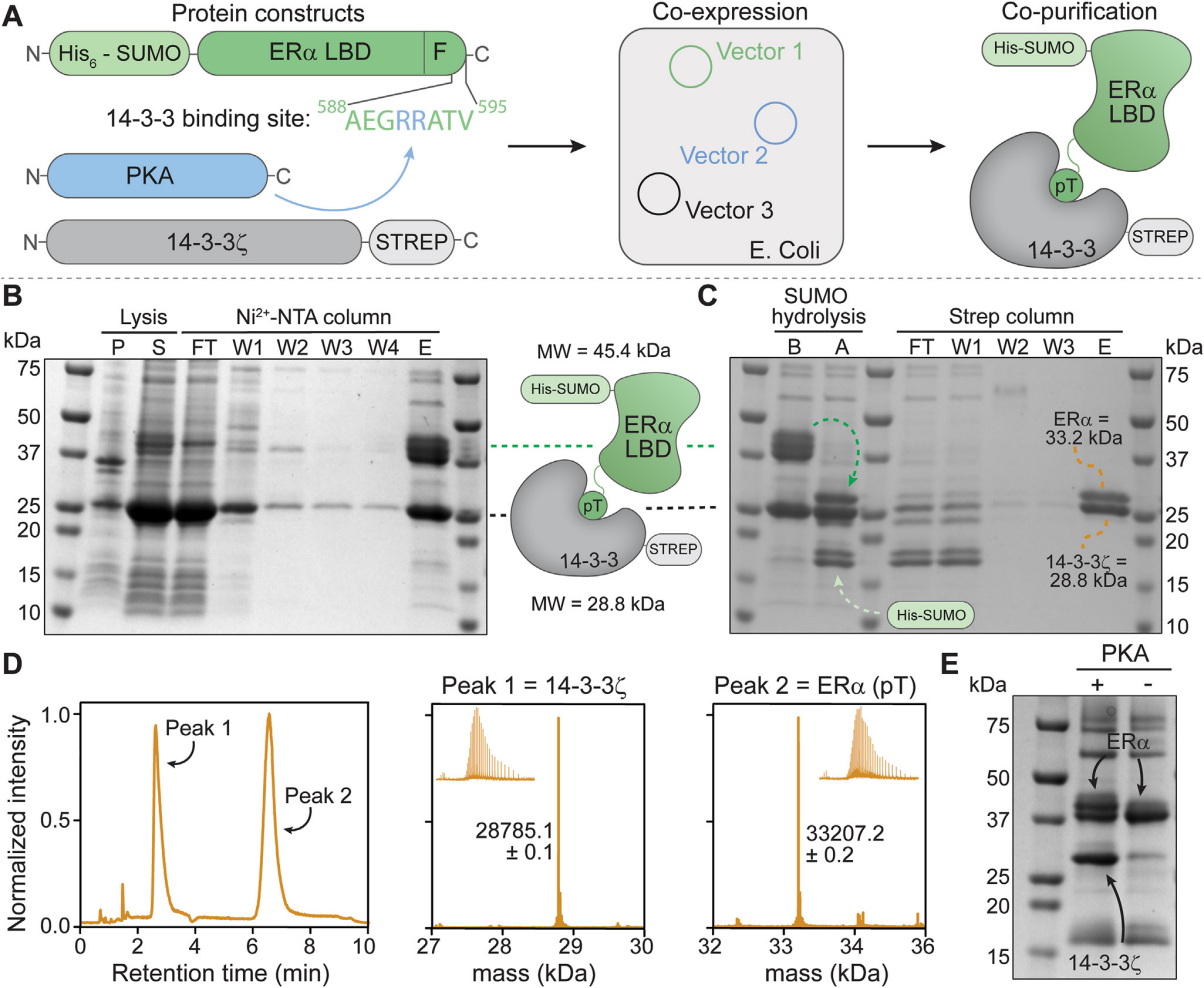
**ERα/14-3-3ζ complex expression and purification.***A*, schematic representation of ERα/14-3-3 co-expression and co-purification. Three protein constructs (*left*) were designed for bacterial expression: (1) His-SUMO-ERα LDB-F domains (*green*) including two mutations to make ERα T594 PKA responsive (*blue*); (2) PKA (*blue*); and (3) 14-3-3ζ-strep (*gray*). These constructs were co-expressed in the same *E. coli* cells (*middle*), leading to the formation of the ERα/14-3-3 complex which was co-purified (*right*). *B* and *C*, SDS-PAGE analysis (Coomassie stain) of the Ni-NTA purification, SUMO-cleavage, and strep purification of the ERα/14-3-3ζ complex. Labels: pellet (P), supernatant (S), flowthrough (FT), wash (W), elution (E), before (*B*), and after (*A*). *D*, QToF-MS analysis of ERα/14-3-3ζ complex including the LC chromatogram and correlated m/z and mass spectra of both peaks corresponding to 14-3-3ζ (expected mass: 28785.1 Da) and phosphorylated ERα (expected mass: 33,207.3 Da). *E*, SDS-PAGE analysis (Coomassie stain) of Ni-NTA purification elution fraction of ERα/14-3-3ζ co-expression in the presence and absence of PKA.

Most biochemical and structural studies on the ERα protein have focused on the ERα LBD, most often ending after helix 12 (∼ residue S554) (57, 58). These studies exclude the 42-residue-long, intrinsically disordered, and solvent-exposed C-terminal F-domain (Fig. S4). The ERα F-domain is described to influence ERα dimerization and co-activator binding; however, as one of the least conserved regions among NRs, the structure and role of the ERα F-domain remain largely unknown (38, 59, 60, 61). The ERα F-domain contains a penultimate threonine residue (T594) that upon phosphorylation facilitates the binding of 14-3-3 to ERα (38). To gain mechanistic insights into the combined role of the ERα LBD and F-domain in concert with 14-3-3 binding, an ERα construct was designed comprising both domains (residues 302–595) (Fig. 2*A*). Since the responsible kinase is not known, native T594 phosphorylation is inaccessible. We thus introduced double-point mutations (F591R/P592R) within the ERα construct, enabling PKA phosphorylation at T594. The two-point mutations make the ERα C-terminal end closely match with the consensus sequence of known PKA substrates (RRXS/TY), thereby increasing the chances of successful phosphorylation of T594 by PKA (62). Fluorescence anisotropy binding studies of 14-3-3ζ to peptide mimics of the wild-type (WT) ERα and PKA-responsive ERα sequence confirmed that the PKA-responsive point mutations did not hamper binding of ERα to 14-3-3ζ (Fig. S5).

The PKA-responsive ERα (ERα(PKA)) construct was expressed and purified from *E. coli*; however, yielded solely in truncated ERα protein (at residues 570 and 572) as apparent from mass spectrometry analysis (Fig. S6). The truncation of the F-domain was addressed by incorporation of a C-terminal strep-tag (ERα(PKA)-strep), resulting in successful expression, purification, and PKA-mediated phosphorylation of ERα (Fig. S7). Notably, the introduction of a C-terminal strep-tag significantly changed the 14-3-3-binding site of ERα. Fluorescence anisotropy binding studies of WT ERα, ERα(PKA), and ERα(PKA)-strep phosphopeptides to 14-3-3ζ showed a 50-fold reduction in binding affinity upon introduction of the strep-tag to the C-terminal end of ERα peptide (Fig. S8). Moreover, stabilization of the ERα(PKA)-strep phosphopeptide/14-3-3 complex by natural product FC-A was significantly reduced. Cooperativity analysis of the ternary 14-3-3/peptide/FC-A complex using WT ERα and ERα(PKA) peptide sequences elicited cooperativity factors (α) of 245 and 366, respectively, whereas the cooperativity factor of FC-A stabilization of the 14-3-3/ERα(PKA)-strep complex was only 15 (Figs. S8 and S9) (63).

Co-crystal structures of 14-3-3 bound to ERα-phosphopeptides revealed why the strep-tagged ERα peptide showed a reduced FC-A responsiveness (Fig. S10). The C-terminal strep-tag occupied the FC-A binding site within the 14-3-3 binding groove, forcing a structural rearrangement of the ERα peptide upon FC-A binding. This makes stabilization by FC-A less favorable compared to the WT ERα peptide. While the ERα(PKA)-strep construct could be used as a representative protein for ERα itself, the protein is less favorable to study 14-3-3 binding and small molecule PPI stabilization. As such, we shifted our focus to an alternative recombinant protein expression approach to obtain the ERα/14-3-3 protein complex.

### Co-expression and co-purification of ERα/14-3-3ζ complex

To circumvent proteolytic cleavage of ERα or the use of C-terminal purification tags, we used bacterial co-expression of ERα, 14-3-3ζ, and PKA to obtain the ERα/14-3-3ζ protein complex. Specifically, N-terminally His-SUMO-tagged ERα(PKA) was expressed together with PKA and strep-tagged 14-3-3ζ in *E. coli* (Fig. 2*A*). Co-expression of 14-3-3ζ and ERα with PKA allowed 14-3-3 to bind *in situ* to the phosphorylated ERα protein shielding the disordered F domain from proteolytic degradation. A similar co-expression approach was previously applied to successfully identify 14-3-3 binding to Ataxin-1 and to obtain a purified Tau/14-3-3 complex (64, 65). Notably, the 14-3-3 protein comprises seven human isoforms which have proven to feature highly similar biochemical and structural features (66, 67). Yeast two-hybrid studies have previously shown that ERα is able to interact with all seven isoforms of 14-3-3 (38). Therefore, we have selected here one 14-3-3 isoform, zeta, as a representative for the class of 14-3-3 proteins. 14-3-3ζ was specifically selected as it expresses well in *E. Coli*, because of its relatively high abundance in the human body (68), and because it has been successfully used in similar biochemical studies of larger 14-3-3 protein complexes, such as the BRAF/14-3-3 complex (50, 51, 69).

The ERα/14-3-3 protein complex was co-purified using three subsequent chromatography methods. First, a Ni-NTA column was used to select for His-tagged ERα protein (Fig. 2*B*). Elution fractions contained both His-tagged ERα and 14-3-3ζ protein, indicating strong binding of 14-3-3ζ to ERα, as 14-3-3 did not contain a His-tag itself. After hydrolysis of the N-terminal His-SUMO tag of ERα, the complex was purified with a streptavidin-column, which again showed co-elution of 14-3-3ζ and ERα (Fig. 2*C*). The ERα/14-3-3ζ protein complex was finally purified by SEC which resulted in the elution of a uniform peak that contained both proteins (Fig. S11). High-resolution mass spectrometry (LC-QToF-MS) analysis showed two protein peaks of which the first corresponded to the mass of 14-3-3ζ and the second to that of the phosphorylated ERα LDB-F protein (Fig. 2*D*). Notably, truncation of ERα was not observed after co-expression and -purification with 14-3-3ζ. Quantitative MS experiments showed the approximately equimolar presence of both 14-3-3ζ and ERα within the purified protein complex sample, indicating a 1:1 binding of the two proteins (Figs. S12 and S13). Furthermore, ERα binding to 14-3-3 proved to be phosphorylation-dependent as co-expression in the absence of PKA did not result in co-elution of 14-3-3ζ with His-tagged ERα (Fig. 2*E*).

### ERα/14-3-3 bind in a 2:2 stoichiometry

Native PAGE analysis of the purified ERα/14-3-3ζ complex showed a single band protein complex indicating stable complex formation between ERα and 14-3-3ζ without the presence of any major excess of either protein (Fig. S14). The addition of a high-affinity competitive 14-3-3 binder (70) disrupted the ERα/14-3-3ζ complex as apparent by two distinct bands that ran in the native PAGE gel at the same heights as 14-3-3ζ and ERα individually, testifying to the stable complex formation between ERα and 14-3-3ζ.

**Figure 3:**
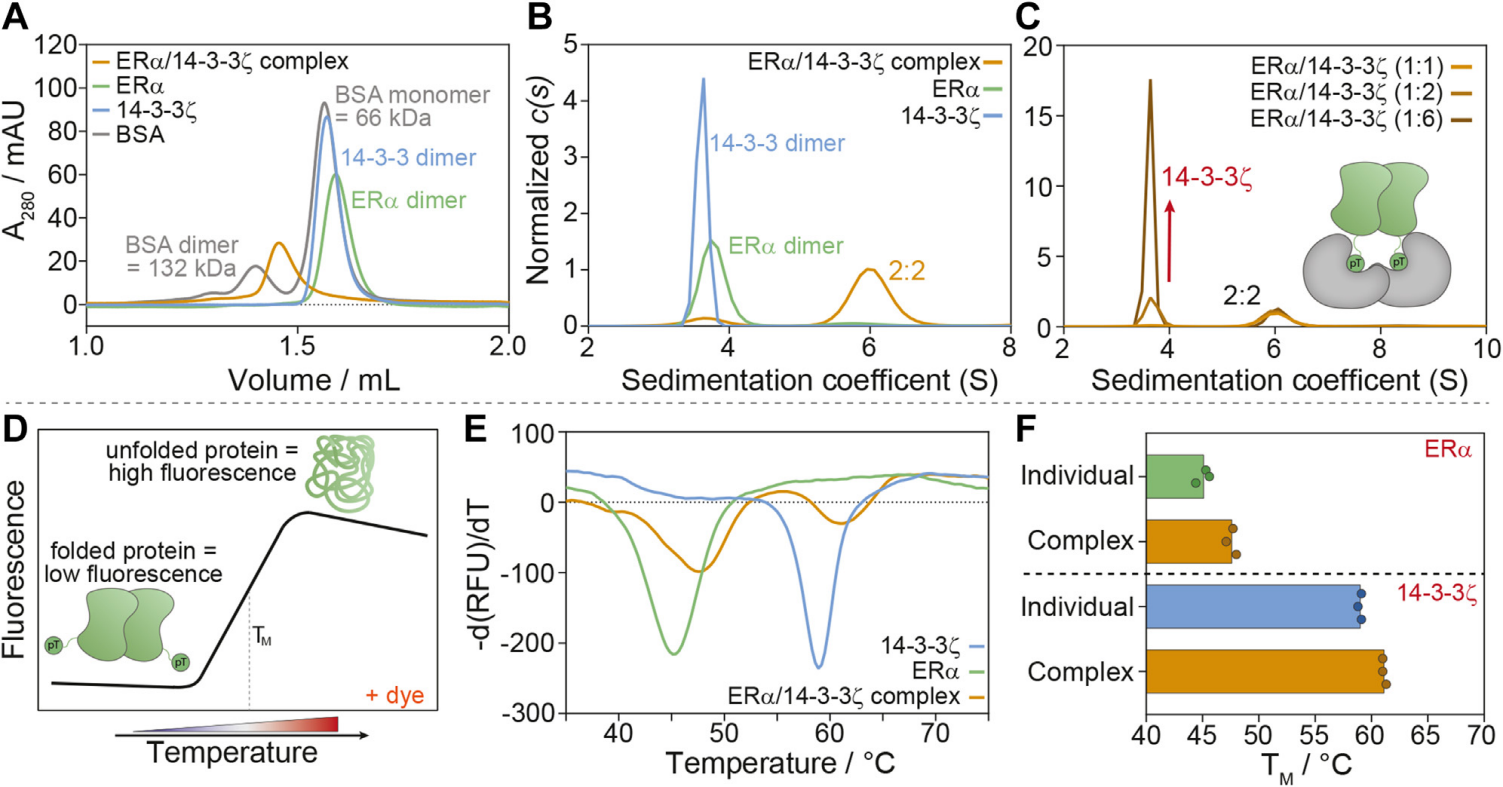
**ERα/14-3-3ζ protein complex characterization.***A*, analytical SEC results of 10 μM BSA (*grey*), 10 μM 14-3-3ζ (*blue*), 10 μM ERα-strep (*green*), and 20 μM ERα/14-3-3ζ protein complex (*orange*) with peak detection based on the absorbance at 280 nm. *B*, area-normalized *c(s)* distributions of 10 μM 14-3-3ζ (*blue*), 10 μM ERα (*green*), and the 30 μM ERα/14-3-3ζ complex (*orange*). *C*, area-normalized *c(s)* distributions of 10 μM ERα/14-3-3ζ complex (1:1) and 10 μM complex with the addition of 5 μM 14-3-3ζ (1:2) or 25 μM 14-3-3ζ (1:6) to compare the effect of different ERα/14-3-3ζ ratios on complex stoichiometry. *D*, schematic representations of differential scanning fluorimetry (DSF) assay where folded proteins are exposed to increasing temperatures which leads to the protein unfolding and subsequent binding of a ProteoOrange dye, resulting in increased fluorescence. *E*, differential melting curve of 5 μM 14-3-3ζ (*blue*), 5 μM ERα (*green*), and 10 μM ERα/14-3-3ζ complex (*orange*). *F*, melting temperatures (T_M_) of ERα and 14-3-3ζ individually and in the complex.

Analytical SEC and sedimentation velocity AUC (SV-AUC) were used as orthogonal approaches to determine the ERα/14-3-3ζ protein complex size and the stoichiometry of ERα and 14-3-3ζ binding (Fig. 3, *A* and *B*). Analytical SEC results showed a single peak for the individual 14-3-3ζ (theoretical M_w_ 29 kDa) and ERα (theoretical M_w_ 33 kDa) proteins which eluted similar to monomeric BSA (theoretical M_w_ 66 kDa), indicating that both proteins themselves were present as stable homodimers in solution (∼60 kDa), which is in line with reported observations (Fig. 3*A*) (59, 67, 71, 72). The ERα/14-3-3ζ complex eluted as a single peak with a clear shift in molecular weight compared to ERα and 14-3-3ζ individually, approaching the dimeric BSA peak around 132 kDa. Together with the quantitative MS studies, which showed the equimolar presence of both ERα and 14-3-3ζ in the purified mixture (Figs. S12 and S13), these results indicated the tetrameric complex formation of an ERα and a 14-3-3ζ homodimer.

SV-AUC sedimentation coefficient distributions *c(s)* validated results observed with SEC. ERα and 14-3-3ζ showed individual peaks with weight-averaged sedimentation coefficients (corrected to 20.0 °C and to the density of water), *s*_*w(20,w*)_, of 4.0 S and 3.8 S, corresponding to M_w_ ∼55 kDa and ∼58 kDa, respectively (theoretical M_w_ of dimeric ERα is 66 kDa; and of dimeric 14-3-3ζ is 58 kDa) (Fig. 3*B*). The ERα/14-3-3ζ complex showed a main peak with a weight-averaged sedimentation coefficient of 6.3 S, corresponding to a M_w_ ∼124 kDa, thus supporting the 2:2 stoichiometry of the ERα:14-3-3ζ complex (72, 73). Notably, a minor peak was observed at peak positions of the 14-3-3ζ and ERα indicating traces of the non-interacting proteins present in the solution under these conditions.

### Dimer-to-dimer binding enhances the ERα/14-3-3 protein complex affinity

Multivalent dimer-to-dimer complex formation was further accessed using a competitive SV-AUC experiment where 14-3-3ζ was added to ERα/14-3-3ζ protein complex to obtain various molar ratios of ERα and 14-3-3ζ. The addition of up to six equivalents of 14-3-3ζ to ERα resulted only in species representing the ERα/14-3-3ζ tetramer and the 14-3-3ζ dimer but did not show any 2:1 14-3-3ζ:ERα complex formation (Fig. 3*C*). This result showed that ERα binds to 14-3-3ζ as a stable dimer. The dimeric state of ERα is hypothesized to cooperatively enhance ERα binding affinity to 14-3-3ζ, similar to earlier described multivalent 14-3-3 binders CFTR and LRRK2 (74, 75). The binding of one ERα monomer to the 14-3-3 dimer brings the second ERα monomer in proximity to 14-3-3, thereby increasing the effective molar concentration of ERα to 14-3-3ζ. Furthermore, dissociation of the protein complex is hypothesized to be slower since two binding interfaces need to dissociate in close succession for 14-3-3ζ and ERα to dissociate. Therefore, dimer-to-dimer binding of ERα and 14-3-3ζ is expected to increase the affinity of ERα for 14-3-3ζ and the stability of the tetrameric protein complex. The dissociation constant K_D_ of dimeric 14-3-3ζ and dimeric ERα was estimated using SV-AUC results of the ERα/14-3-3 complex (Fig. S15). Based on the area under the curve, the amount of ERα and 14-3-3ζ in complex and alone was quantified from which the K_D_ was calculated using the steady state equilibrium binding equation (K_D_ = [A][B]/[AB]). This calculation provided a K_D_ ∼32 ± 6 nM. This affinity is almost 10-fold higher than that of the ERα phosphopeptide binding to 14-3-3, indicating stronger binding of the phosphorylated ERα LBD-F domain protein due to the dimer-to-dimer binding mechanism.

### Complex formation increases the thermal stability of both protein partners

The ERα/14-3-3ζ complex stability was studied using differential scanning fluorimetry (DSF) studies (Fig. 3*D*). ERα and 14-3-3ζ alone showed melting temperatures of 45.2 °C ± 0.8 deg. C and 59.0 °C ± 0.2 deg. C, respectively (Fig. 3, *E* and *F*). ERα phosphorylation at T594 did not influence its melting temperature (Fig. S16). The ERα/14-3-3ζ protein complex showed two distinct melting peaks (Fig. 3*E*), with ∼2 °C increased thermal stability when compared to the individual protein partners (Fig. 3*F*). A similar increase in thermal stability for 14-3-3ζ was observed upon the addition of an ERα phosphopeptide (Fig. S16). These results thus show a mutual stabilizing effect of 14-3-3ζ and ERα upon tetramer formation.

### Mapping of interactions between ERα and 14-3-3ζ using HDX-MS

**Figure 4:**
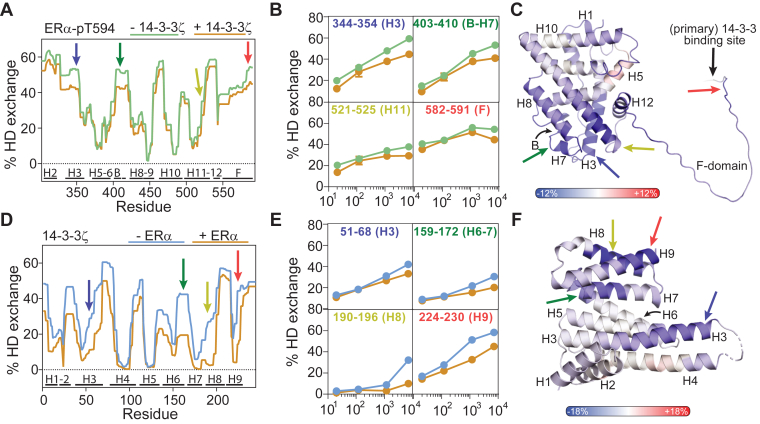
**Hydrogen-deuterium exchange (HDX) of ERα/14-3-3ζ protein complex.***A*, HDX-MS exchange profile of ERα-pT594 (*green*) and ERα in ERα/14-3-3ζ protein complex (*orange*) after 2 h of HDX. *B*, time-dependent HDX profile of individual ERα peptides. *C*, HDX difference profile of ERα-pT594 in the ERα/14-3-3ζ complex minus ERα-pT594 alone after 2 h incubation which displays the effect of 14-3-3ζ binding on deuterium exchange in ERα. Shielding effects (less deuterium exchange) are shown in *blue* and deshielding effects (increase in deuterium exchange) in *red*. Results are displayed on the predicted ERα Alphafold structure (LBD-F domains). *D*, HDX-MS exchange profile of 14-3-3ζ (*blue*) and 14-3-3ζ in ERα/14-3-3ζ protein complex (*orange*) after 2 h of HDX. *E*, time-dependent HDX profile of individual 14-3-3ζ peptides. *F*, HDX difference profile of 14-3-3ζ in the ERα/14-3-3ζ complex minus 14-3-3ζ alone after 2 h incubation which displays the effect of ERα binding on deuterium exchange in 14-3-3ζ. Results are displayed on the 14-3-3ζ crystal structure, PDB: 6F09.

Hydrogen-deuterium exchange (HDX-MS) assays were performed on the individual proteins 14-3-3ζ and ERα, and the ERα/14-3-3ζ protein complex, to identify which regions within the ERα and 14-3-3ζ protein were involved in protein complex formation (Figs. 4 and S17–S20). The proteins were incubated in deuterium-containing buffers which were quenched after 20 s, 2 min, 20 min, and 2 h. Proteins were digested using pepsin after which the amount of deuteration of individual peptides was determined using mass spectrometry. HD exchange kinetics of ERα was followed for 248 peptides, covering 98.6% of the protein sequence, and 240 peptides for 14-3-3ζ which cover 100% of the sequence.

An HD exchange profile was made to visualize the deuteration kinetics of each ERα residue for *apo*-ERα and ERα within the ERα/14-3-3ζ complex (Figs. 4*A* and S17). Fast deuterium exchange kinetics (>30% deuteration after 20 s) were mainly observed in helix 1 to 2 (H1-2), the beta sheets (B), helix 7 (H7), residues between helix 9 and 10, helix 12 (H12), and the entire F-domain. Residues in H5-6, H8-9, H10, and H11, on the other hand, showed low amounts of deuterium exchange. These results corresponded nicely with the ERα crystal structures where regions with fast deuterium exchange kinetics are typically present in flexible and/or solvent-exposed regions of ERα. Notably, the C-terminal F-domain of ERα has never been crystallized before or studied with alternative structural biology techniques. Within this study, fast HD exchange kinetics were observed within the entire F-domain indicating that the ERα F-domain is most probably unstructured and solvent-exposed, as observed in the predicted Alphafold structure (76, 77).

14-3-3ζ binding to ERα decreased the deuteration kinetics of several regions within the ERα protein (Fig. 4, *A*–*C*; Figs. S17 and S18). Shielding effects were determined by calculating the difference in HDX between 14-3-3ζ-bound ERα and ERα by itself, after which this difference profile was visualized on the ERα Alphafold structure (Fig. 4*C* and S17). Most pronounced shielding effects were observed in the N-terminal side of H3 (residues 328–354), the beta sheets through H7 (residues 397–420), the C-terminal side of H11 (residues 521–528), and the C-terminal end of the F-domain (residues 583–591) (Fig. 4, *A*–*C*; Figs. S17 and S18). Smaller effects were observed in H1-2 (residues 302–327), H8 (residues 421–444), H12, and the F-domain (residues 531–571). H4-6, H9-10, and the N-terminal part of H11 seemed unaffected, although it should be mentioned that these regions showed minor deuteration in the first place. Interestingly, although the 14-3-3 binding groove is known to primarily bind the C-terminal end of the ERα F-domain, multiple regions additional to the F-domain seem to be affected by 14-3-3 binding. Shielding effects were observed for almost all flexible and solvent-exposed regions within ERα, indicating an overall stabilization of the ERα fold upon tetramer formation. Most pronounced effects were clustered on the ‘bottom’ of the ERα LBD structure (H7, H3 N-term, H11 C-term), indicating the proximity of 14-3-3ζ to this side of ERα, albeit with the dynamic movement of the ERα LBD dimer, facilitated by the long and flexible F-domain, leading to mild shielding effects at all sides of the ERα LDB. Notably, ERα/14-3-3ζ dimer-to-dimer binding might result in differential binding of one ERα monomer in comparison to the other ERα monomer, potentially further explaining why many regions within the ERα protein are mildly affected upon 14-3-3ζ binding.

Deuteration kinetics for 14-3-3ζ in the absence and presence of ERα protein were similarly analyzed (Fig. 4, *D*–*F*, Figs. S19 and S20). The regions of high and low exchange rates of 14-3-3ζ alone corresponded well to known crystal structures and previously published HDX of 14-3-3 (78, 79), with high deuteration in loops between H2-3, H3-4, H4-5, and H8-9, and none to minor amounts of deuteration in H2, H3, H4, H5, H7, and H9. ERα binding to 14-3-3ζ led to shielding effects on various parts of the 14-3-3ζ protein, which was most pronounced after 2 h of incubation (Fig. 4, *D*–*F*; Figs. S19 and S20). The largest shielding effects were observed within peptides in the C-terminal end of H3 (residues 50–68), H6-7 (residues 154–174), H8 (residues 180–199), and the C-terminus in H9 (residues 217–230). These shielded regions strongly correlated with the 14-3-3 binding groove (H3, H7 and H9) where the ERα C-terminus binds. Interestingly, also H8 on “top” and H6 on the “back” of the 14-3-3 protein were partially shielded, indicating that these 14-3-3 regions are potentially in close proximity to other parts of ERα. In contrast, the “base” of the 14-3-3 protein seems to be less affected by ERα binding, indicating the absence of direct contact with ERα (Fig. S19). Crystallography and HDX studies of other 14-3-3 PPIs typically show similar binding interfaces involving the ‘top’ of 14-3-3 (H8-9), whereas the “base” of 14-3-3 is not involved with the PPI formation (48, 69, 79, 80, 81).

### 14-3-3ζ can bind the ERα Y573S drug-resistant mutant

Point mutations in the ERα LBD are known to modulate ERα conformation and transcriptional activity. ERα Y537S is one of the most prevalent somatic mutations in patients with breast cancer, typically acquired after antiestrogen treatment (54). Structural and biophysical characterization has shown that the Y537S mutation places H12 in an agonistic, constitutively active, conformation (Fig. S21), causing this ERα mutant to be resistant to antiestrogen treatment (54, 82). Therefore, it is of high interest to study 14-3-3ζ binding to ERα-Y537S as it may provide a new approach to modulate the transcriptional activity of the drug-resistant ERα-Y537S mutant. The ERα-Y537S/14-3-3ζ complex could successfully be co-expressed and co-purified using the aforementioned methodologies for WT ERα/14-3-3ζ complex purification, indicating that the Y537S point mutation did not impede 14-3-3 binding. SV-AUC confirmed this as similar sedimentation distributions were obtained for the ERα-Y537S/14-3-3ζ complex and the WT ERα/14-3-3ζ protein complex (Fig. S22*A*). Furthermore, DSF studies showed a ∼2 °C enhancement of thermal stability of both ERα-Y537S and 14-3-3ζ upon complex formation, similar to WT ERα (Fig. S22, *B*–*D*). Interestingly, ERα-Y537S in the absence of 14-3-3 showed melting temperatures of 47.1 °C ± 0.5 deg. C, which is ∼2 °C higher than wildtype ERα, indicating higher thermal stability of ERα when mutated. All data together confirmed ERα-Y537S/14-3-3ζ complex formation, providing an interesting entry point of targeting this mutant *via* its interaction with 14-3-3.

### ERα ligand binding is independent of ERα/14-3-3 complex formation

**Figure 5:**
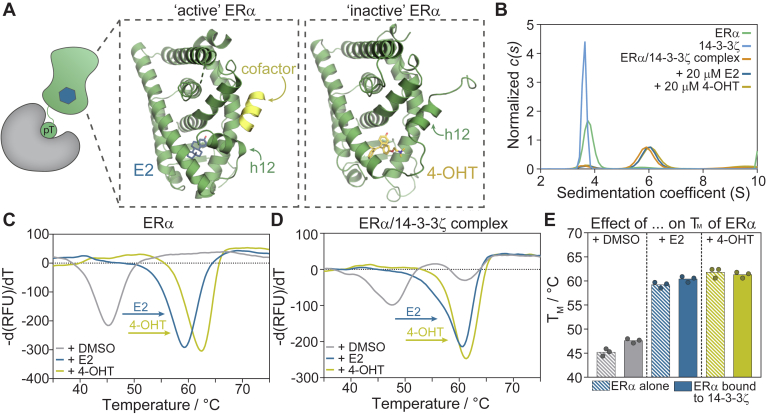
**ERα ligand binding to the ERα/14-3-3ζ complex.***A*, schematic representation of ERα/14-3-3 complex with ERα ligand (*blue hexagon*) binding in the ERα LBD. Crystal structures of ERα ligands E2 and 4-OHT bound to the ERα ligand binding domain (*green cartoon*). Ligand binding changes the conformation of helix 12 and allows, in case of E2, to cofactor peptide binding (*yellow cartoon*), PDB: 5WGD & 3ERT. *B*, area-normalized sedimentation distributions *c(s)* of 10 μM 14-3-3ζ (*blue*), 10 μM ERα (*green*), and 10 μM ERα/14-3-3ζ complex (*orange*). The latter was also analyzed in the presence of 20 μM E2 (*blue*) or 20 μM 4-OHT (yellow). *C* and *D*, differential melting curves of 5 μM ERα (*C*) or 10 μM ERα/14-3-3ζ protein complex (*D*) in the presence of DMSO (negative control), 100 μM E2 or 100 μM 4-OHT. *E*, melting temperatures T_M_ of ERα by itself or when bound to 14-3-3ζ in the presence of DMSO, E2, or 4-OHT.

Small molecule ligands play an important role in the regulation of ERα transcriptional activity in both healthy and diseased state, making it highly valuable to study their effects on ERα/14-3-3 complex formation. Therefore, we set out to study the effect of ERα/14-3-3 complex formation on ligand binding to both WT and the Y537S mutant of ERα. Here, we specifically studied the endogenous ERα agonist E2 and therapeutic partial antagonist 4-OHT (Fig. 5*A*) (83, 84). In both SV-AUC and native PAGE, E2 and 4-OHT did not show any effect on the ERα/14-3-3 protein complex size, apparent by the similar *c(s)* distribution of the ERα/14-3-3ζ complex in the presence and absence of ligands (Figs. 5*B* and S23). Furthermore, ligand-dependent DSF studies were performed to determine the effect of ERα ligand binding on the thermal stability of ERα in complex with 14-3-3ζ. (Fig. 5, *C*–*E*; Figs. S24 and S25). The addition of E2 or 4-OHT to both phosphorylated and non-phosphorylated ERα strongly increased the ERα melting temperature by 14 °C and 16 °C, respectively (Figs. 5*C* and S24). Similarly, E2 or 4-OHT addition to the ERα/14-3-3ζ complex resulted in a clear shift of the ERα T_M_ from 47.6 to 60.4 °C and 61.3 °C, respectively (Figs. 5, *D*–*F* and S24). Similar effects of E2 and 4-OHT were observed for experiments with the ERα-Y537S/14-3-3ζ complex (Fig. S25). Overall, the SV-AUC and DSF data indicated that ERα ligands did not disrupt ERα/14-3-3ζ complex formation and ERα was fully ligand responsive when bound to 14-3-3ζ.

### E2 induces the active conformation of ERα also when bound to 14-3-3

**Figure 6:**
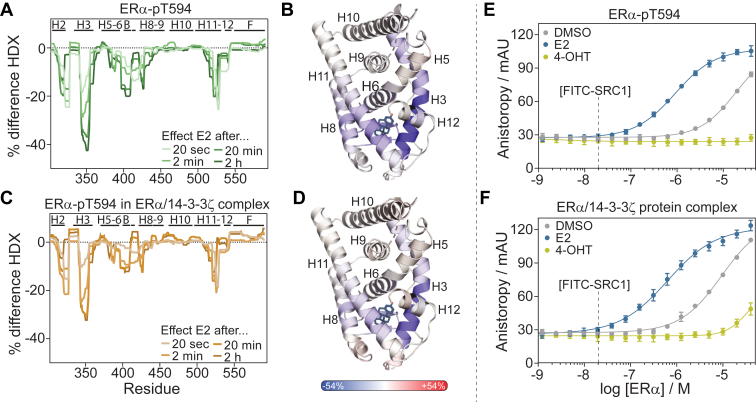
**Effect of ERα ligand binding to ERα/14-3-3ζ complex on ERα conformation and co-factor binding.***A* and *C*, difference HDX exchange profile of ERα-pT594 (*A*) and ERα-pT594 in ERα/14-3-3ζ protein complex (*C*) upon E2 binding after 20 s, 2 min, 20 min or 2 h of HDX. *B* and *D*, HDX difference profile of ERα-pT594 (*B*) and ERα in ERα/14-3-3ζ protein complex (*D*) upon addition of E2 ligand after 2 h incubation. The shielding effect (less deuterium exchange) is shown in *blue* and deshielding effects (increase in deuterium exchange) in *red*. Results are displayed on the ERα LBD crystal structure, PDB: 5WGD. *E* and *F*, fluorescence anisotropy SRC-1 co-factor recruitment assay where ERα-pT594 or ERα/14-3-3ζ protein complex was titrated to 20 nM FITC-labeled SRC-1 peptide in the presence of DMSO (negative control), 100 μM E2 or 100 μM 4-OHT.

The effect of E2 on the conformation of ERα when bound 14-3-3 was determined using HDX experiments. The effect of E2 on each ERα residue was determined by subtracting the HDX exchange profiles of ERα with and without ligand. Interestingly, E2 binding seemed to affect similar regions in ERα alone and ERα bound to 14-3-3ζ (Figs. 6, *A*–*D* and S26–S30). Major shielding effects were observed for parts of H3 (residues 341–361), the beta sheet region with adjacent loops (residues 398–410), and the C-terminal part of H11 (512–527). Smaller effects were also observed in H1-2 (residues 314–320), H6 (residues 380–396), H8 (residues 421–438), the H11 to 12 loop, and H12 (residues 528–541). No E2-induced differences were observed around H5, H9, H10, and the F-domain of ERα. Notably, the overall effects of E2 appeared to be smaller (max. shielding effect ∼30% instead of 40%) when ERα was bound to 14-3-3ζ which can be explained by the partial shielding effects that 14-3-3ζ already has on ERα, making the effect of E2 less pronounced. The affected regions of ERα upon E2 binding correlated well with published ERα-E2 crystal structures (Fig. 6, *B* and *D*), where E2 binds in a pocket formed by H3, H6, H8, the beta sheets, and H11 to 12 (84). Interestingly, these results indicated that the ERα F-domain conformation was not significantly affected by E2 binding, which has not been studied on a structural level previously. The similar shielding effects upon E2 binding to ERα alone and the ERα/14-3-3ζ complex, clearly showed that 14-3-3ζ does not influence the E2-induced conformational changes in ERα. This suggests that the ERα F-domain is sufficiently long and flexible to accommodate ligand-induced conformational changes to helix 12, without affecting 14-3-3 binding to ERα.

### ERα-cofactor binding is permitted when 14-3-3 is bound to ERα

Next, we sought to understand how 14-3-3 influences ERα cofactor recruitment. ERα binding to a cofactor was studied in FA experiments in which ERα was titrated, in the absence and presence of 14-3-3ζ, against a fluorescein-labeled peptide representing an alpha-helical LXXLL recognition motif from the p160 family member SRC-1/NCoA-1 (Fig. 6, *E* and *F*; Fig. S31) (59). These ERα-cofactor binding studies were furthermore performed in the presence of agonist ligand E2 or antagonistic ligand 4-OHT. Both non-phosphorylated and phosphorylated ERα showed typical binding curves to the LXXLL peptide (K_D_ ∼ 20 μM), which was strongly enhanced upon the addition of E2 (K_D_ = 1.0 ± 0.2 μM) and abolished in the presence of 4-OHT (Figs. 6*E* and S31). Titration of the ERα/14-3-3ζ complex showed similar binding curves as observed for ERα alone. ERα, in complex with 14-3-3ζ, bound SRC-1 peptide with a K_D_ of 9.6 ± 1.1 μM, and featured a strong E2 responsiveness (K_D_ = 0.6 ± 0.1 μM) (Figs. 6*F* and S31). Furthermore, 4-OHT strongly reduced ERα binding to the LXXLL peptide, when ERα was bound to 14-3-3ζ.

FA assays with the mutated ERα-Y537S construct showed improved cofactor recruitment of *apo* ERα-Y537S in comparison to WT ERα (Fig. S31). *Apo* ERα-Y537S provided a binding affinity for the LXXLL peptide of 1.7 ± 0.1 μM, which was almost similar to the amount of cofactor recruitment in the presence of agnostic ligand E2 (K_D_ = 0.8 ± 0.1 μM). These results align with previously published data where the Y537S mutation resulted in a constitutively active conformation of ERα in the absence of agonistic ligands (54). Furthermore, ERα-Y537S was found to be less sensitive to 4-OHT inhibition of cofactor recruitment (Fig. S31). Similar to WT ERα, 14-3-3 binding did not significantly influence SRC-1 recruitment to the ERα-Y537S protein (Fig. S31).

### ERα/14-3-3 PPI stabilization by FC-A is orthogonal to ERα ligand binding

**Figure 7:**
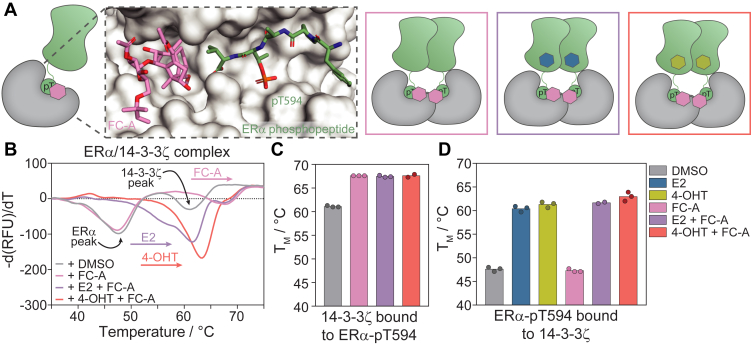
**ERα/14-3-3ζ complex stabilization by FC-A.***A*, schematic representation of FC-A (*pink hexagon*) binding to the ERα/14-3-3 protein interface with the co-crystal structure of 14-3-3σ (*gray surface*) with the Erα-derived C-terminal phosphopeptide (*green sticks*) and the stabilizing molecule Fusicoccin-A (FC-A, *pink sticks*) and PDB: 4JDD. *B*, differential melting curve of 10 μM ERα/14-3-3ζ protein complex in the presence of DMSO (negative control), 100 μM FC-A, 100 μM FC-A, and 100 μM E2 and, 100 μM FC-A and 100 μM 4-OHT. *C*, bar plot of melting temperatures of 14-3-3ζ in the ERα/14-3-3 complex from differential melting curves in panel b. *D*, bar plot of melting temperatures of ERα in the ERα/14-3-3 complex from differential melting curves in panel b and Fig. S24.

The natural product FC-A is a known stabilizer of the ERα/14-3-3ζ PPI. This small molecule binds at the interface of ERα/14-3-3 protein complex (Fig. 7*A*) and thereby increases the affinity between the binding partners (38, 46). DSF studies were used to determine the effect of FC-A on 14-3-3ζ, ERα, or the ERα/14-3-3ζ protein complex, for both WT ERα and ERα-Y537S (Fig. 7*B*; Figs. S24 and S25). As expected, FC-A had no effect on the T_M_ of 14-3-3ζ or ERα alone (Fig. S24) but increased the T_M_ of the 14-3-3ζ protein in complex with WT ERα from 61.1 to 67.6 °C (+6.5 °C) (Fig. 7, *B* and *C*). Similarly, FC-A was found to stabilize the ERα-Y537S/14-3-3ζ complex as apparent from the increase 14-3-3ζ melting temperature from 60.2 to 66.3 °C (+6.1 °C) (Fig. S25). FC-A did not affect the T_M_ of the ERα protein, in the ERα/14-3-3ζ complex, indicating a local effect of FC-A, confined to the composite binding pocket. This is in line with the previous observations, where the ERα F-domain acts as a long and flexible linker between the most C-terminal ERα residues binding in the 14-3-3 binding groove, and the globular ERa LBD dimer.

Interestingly, the FC-A-induced increase of 14-3-3ζ thermal stability was fully orthogonal to E2 or 4-OHT binding to both the WT ERα and the Y537S mutant. In the presence of E2 and 4-OHT, FC-A still increased the melting temperature of 14-3-3ζ in the ERα/14-3-3ζ protein complex with +6.4 °C and +6.5 °C, respectively (Figs. 7*C* and S24). In reverse, the earlier described increase in ERα melting temperature upon the addition of E2 and 4-OHT (Fig. 5, *D* and *E*) was not influenced by ERα/14-3-3ζ stabilization by FC-A (Figs. 7*D* and S24). E2 and 4-OHT increased the ERα T_M_, in the ERα/14-3-3ζ complex, with +12.8 °C and +13.7 °C, respectively, which was even slightly increased in the presence of FC-A (+14.1 °C E2; +15.4 °C 4-OHT) (Figs. 7*D* and S24). FC-A thus clearly stabilized the ERα/14-3-3ζ complex, for both WT ERα and Y537S mutant, and showed to be independent of ERα ligand binding.

## Discussion

NR drug discovery approaches have mainly focused on targeting the NR endogenous ligand binding pocket present within the LBD. Despite great successes using this approach, significant interest has developed in alternative manners to modulate NRs. An orthogonal entry for NR modulation is offered by their PPIs with the 14-3-3 protein. However, the highly relevant molecular understanding of these NR/14-3-3 PPIs is often lacking, while this is necessary to identify new entry points for NR drug discovery. Here we studied the NR ERα and its interaction with 14-3-3 on a molecular level. Co-purification of the intact complex of 14-3-3ζ and the ERα LBD and F domains revealed high-affinity binding between ERα and 14-3-3ζ *via* the formation of a tetrameric complex between an ERα homodimer and a 14-3-3ζ homodimer. Furthermore, the binding of 14-3-3ζ to the disease-relevant Y537S-ERα was confirmed, highlighting the possibility of targeting the ERα/14-3-3ζ PPI as an alternative drug discovery approach for drug-resistant mutants of ERα.

Both agonist (E2) and antagonist (4-OHT) binding to the ERα LBD did not disrupt 14-3-3 binding to ERα, as apparent from SV-AUC and the native PAGE. Furthermore, natural ligand E2 induced activating conformational changes of the ERα LBD and subsequent cofactor peptide recruitment, in a similar fashion for ERα in isolation and ERα in complex with 14-3-3ζ. Similarly, synthetic ligand 4-OHT showed antagonistic behavior for ERα alone and ERα in complex with 14-3-3ζ, as observed by reduced cofactor recruitment. Finally, the ERα/14-3-3ζ PPI stabilization by FC-A was shown to be functional and not impeded, nor dependent, on ERα ligand binding in both wild-type ERα and the Y537S mutated protein.

Combined, these results inform that 14-3-3ζ binding to ERα, and stabilization of this PPI by FC-A, function independently of conformations, mutations, and liganded state of the ERα LBD. This orthogonality is most likely facilitated by the long and flexible 42-residue F-domain of ERα, accommodating ERα conformations to not affect 14-3-3 binding. Molecular stabilization of the ERα/14-3-3 protein complex with molecular glues like FC-A would therefore be a potential entry point for targeting ERα and its drug-resistant variants. The orthogonality of the molecular events within the ERα would even bode for dual targeting of both the PPI interface and the classical ERα ligand binding pocket. Furthermore, whereas orthosteric drugs such as 4-OHT also show binding to ERβ (85) and ERRγ (86), next to ERα, 14-3-3ζ binding to ERα occurs at the ERα-unique C-terminus, providing the possibility to target ERα in a highly selective manner.

The concept of therapeutic targeting of the ERα/14-3-3 PPI could be envisioned to be translatable to other NR/14-3-3 protein complexes. So far, 14-3-3 has been identified as the binding partner of eight NRs, for which 14-3-3 binds to each NR in a unique manner. The NR/14-3-3 interactions form a potential entry point for targeting ‘hard-to-drug’ NRs due to, for example, drug resistance or the absence of an orthosteric pocket in the LBD. A potential example is the Androgen Receptor (AR), an established prostate cancer target. Although prostate cancer is initially often successfully targeted with androgen deprivation therapy or AR antagonists such as enzalutamide, drug- and castration-resistant AR mutants or splice variants are often developed within patients with prostate cancer (25, 87, 88). The most prevalent AR splice variant, AR-V7, even lacks the entire LBD while remaining constitutively active, making it extremely challenging to target this drug-resistant variant of AR (87, 88). The binding of 14-3-3 to the NTD of AR, which remains present in the AR splice variants, provides an alternative entry point of targeting AR in drug- and castration-resistant patients with prostate cancer. In all cases, mechanistic and structural insights into the formation of the NR/14-3-3 complex, such as those obtained in this study for the ERα/14-3-3 complex are urgently needed.

## Experimental procedures

### Protein expression and purification - ERα-strep

A His-Sumo-ERαLBD-F-strep (residues 302–595 in pCDFDuet-1, uniprot P03372) construct was designed which contained three mutations (S305A, F591R, and P592R) to introduce a PKA recognition motif for *in vitro* phosphorylation of T594, and to remove a potential PKA phosphorylation site S305. pCDFDuet-1-His-SUMO-ERaLBD-F-strep was transformed into NiCo21 (DE3) competent cells using heat stock and were plated on LB-agar plates containing 100 μg/ml streptomycin. A single colony was selected and cultured overnight in 50 ml LB medium (100 μg/ml streptomycin) at 37 °C. After overnight incubation, cultures were transferred to 2 l ZYP-5052 medium (100 μg/ml streptomycin) and incubated at 37 °C, 180 rpm until an OD_600_ of ∼2 was reached. Protein expression was then induced with 0.4 mM isopropyl-β-d-thiogalactoside (IPTG), and cultures were incubated overnight at 18 °C, 180 rpm. Cells were harvested by centrifugation (8600 rpm, 20 min, 4 °C) and resuspended in lysis buffer (10 ml/g pellet; 20 mM Tris, pH 7.5, 300 mM NaCl, 10 mM imidazole, 5 mM MgCl_2_, 5% (v/v) glycerol, 2 mM beta-mercaptoethanol (βME) containing cOmplete EDTA-free Protease Inhibitor Cocktail tablets (1 tablet/100 ml lysate) and Benzonase Nuclease (Milipore, 5 μl/100 ml). The cell suspension was lysed using an Emulsiflex-C3 homogenizer (Avestin) and the cell lysate was cleared by centrifugation (20,000 rpm, 30 min, 4 °C). Protein-containing supernatant was loaded on 2 × 5 ml Ni-NTA columns (HisTrap column, Cytiva) at 5 ml/min and 4 °C. Columns were washed with 3 × 5 CV Wash Buffer (20 mM Tris, pH 7.5, 300 mM NaCl, 25 mM imidazole, 2% (v/v) glycerol, 2 mM βME) until proteins concentrations dropped below 0.05 mg/ml. His-SUMO-ERαLBD-F-strep was eluted using 2 to 3 CV Elution Buffer (20 mM Tris, pH 7.5, 300 mM NaCl, 500 mM imidazole, 2% (v/v) glycerol, 2 mM βME). His-SUMO-ERαLBD-F-strep containing elution fractions were subsequently incubated with SUMO hydrolase (1:100) to remove the His-SUMO tag from ERα during o/n dialysis at 4 °C (20 mM Tris, pH 7.5, 150 mM NaCl, 10 mM MgCl_2_, 5% (v/v) glycerol, 2 mM βME). SUMO-cleaved ERαLBD-F-strep was loaded on a 5 ml Streptavidin column (Strep-TactinXT 4Flow cartridge, IBA) at 3 ml/min. Columns were washed with Buffer W (100 mM Tris pH 8.0, 150 mM NaCl, 1 mM EDTA) collecting 2 CV until protein concentrations dropped below 0.05 mg/ml. ERαLBD-F-strep was eluted using 4 CV Buffer BXT (100 mM Tris pH 8.0, 150 mM NaCl, 1 mM EDTA, 50 mM biotin). Finally, ERαLBD-F-strep was purified by SEC (Superdex 75pg Hiload 16/600) at 1 ml/min and 4 °C using SEC buffer (20 mM Tris pH 7.5, 150 mM NaCl, 10 mM MgCl_2_, 0.5 mM TCEP). Protein-containing fractions were combined and concentrated to >5 mg/ml, aliquoted, and stored at −80 °C. Correct mass and purity of the proteins were confirmed using SDS-PAGE and Q-ToF LC-MS analysis.

Phosphorylated ERα (ERα-pT594-strep) was expressed and purified in a similar manner as described earlier for non-phosphorylated ERα-strep. An *in vitro* phosphorylation was additionally included directly after SUMO cleavage, and before purification on the streptavidin and SEC columns. SUMO-cleaved ERαLBD-F-strep was phosphorylated by incubation with in house-expressed His-PKA-strep (in a 1:50 ratio PKA:substrate) and 500 μM ATP, for 3 h at 4 °C.

### Protein expression and purification–14-3-3ζ-strep

A 14-3-3zeta-strep (residues 1–245 in pETDuet-1, uniprot P63104) construct was transformed into NiCo21 (DE3) competent cells using heat stock and was plated on LB-agar plates containing 100 μg/ml ampicillin. A single colony was selected and cultured overnight in 8 ml LB medium (100 μg/ml ampicillin) at 37 °C. After overnight incubation, cultures were transferred to 500 ml TB medium (100 μg/ml ampicillin) and incubated at 37 °C, 180 rpm until an OD_600_ of 0.6 to 1.2 was reached. Protein expression was induced with 0.4 mM IPTG, and cultures were incubated overnight at 18 °C, 180 rpm. Cells were harvested by centrifugation (8600 rpm, 20 min, 4 °C) and resuspended in lysis buffer (10 ml/g pellet; 20 mM Tris, pH 7.5, 300 mM NaCl, 10 mM imidazole, 5 mM MgCl_2_, 5% (v/v) glycerol, 2 mM βME) containing cOmplete EDTA-free Protease Inhibitor Cocktail tablets (1 tablet/100 ml lysate) and Benzonase Nuclease (Milipore, 5 μl/100 ml). The cell suspension was lysed using an Emulsiflex-C3 homogenizer (Avestin), and the cell lysate was cleared by centrifugation (20,000 rpm, 30 min, 4 °C). Protein-containing supernatant was loaded on a 5 ml Streptavidin column (Strep-TactinXT 4Flow cartridge, IBA) at 3 ml/min. Columns were washed with 4x 2CV Buffer W (100 mM Tris pH 8.0, 150 mM NaCl, 1 mM EDTA). 14-3-3ζ-strep was eluted using 2 CV Buffer BXT (100 mM Tris pH 8.0, 150 mM NaCl, 1 mM EDTA, 50 mM biotin). Finally, 14-3-3ζ-strep was purified by SEC (Superdex 75pg Hiload 16/600) at 1 ml/min and 4 °C using SEC buffer (20 mM Tris pH 7.5, 150 mM NaCl, 10 mM MgCl_2_, 0.5 mM TCEP). Protein-containing fractions were combined and concentrated to >5 mg/ml, aliquoted, and stored at −80 °C. Correct mass and purity of the protein were confirmed using SDS-PAGE and Q-ToF LC-MS analysis.

### Protein expression and purification—ERα/14-3-3ζ protein complex

His-SUMO-ERαLBD-F (residues 302–595 in pCDFDuet-1, uniprot P03372) was co-transformed with full-length 14-3-3zeta (residues 1–245 in pETDuet-1, uniprot P63104) and SUMO-PKA (residues 1–351 in pACYC, UniProt P17612) (see all protein sequences in Supporting information) into NiCo21 (DE3) competent cells using heat stock and were plated on LB-agar plates containing antibiotics for selection: 100 μg/ml streptomycin, 100 μg/ml ampicillin and 25 μg/ml chloramphenicol). A single colony was selected and cultured overnight in 50 ml LB medium (containing the same antibiotics) at 37 °C. After overnight incubation, cultures were transferred to 2 l ZYP-5052 medium (containing the same antibiotics) and incubated at 37 °C, 180 rpm until an OD_600_ of ∼2 was reached. Protein expression was induced with 0.4 mM IPTG and cultures were incubated overnight at 18˚C, 180 rpm. Cells were harvested by centrifugation (8600 rpm, 20 min, 4 °C) and resuspended in lysis buffer (10 ml/g pellet; 20 mM Tris, pH 7.5, 300 mM NaCl, 10 mM imidazole, 5 mM MgCl_2_, 5% (v/v) glycerol, 2 mM βME) containing cOmplete EDTA-free Protease Inhibitor Cocktail tablets (1 tablet/100 ml lysate) and Benzonase Nuclease (Milipore, 5 μl/100 ml). The cell suspension was lysed using an Emulsiflex-C3 homogenizer (Avestin) and the cell lysate was cleared by centrifugation (20,000 rpm, 30 min, 4 °C). Protein-containing supernatant was loaded on 2 × 5 ml Ni-NTA columns (HisTrap column, Cytiva) at 5 ml/min and 4˚C. Columns were washed with Wash Buffer (20 mM Tris, pH 7.5, 300 mM NaCl, 25 mM imidazole, 2% (v/v) glycerol, 2 mM βME) collecting each time 1 to 3 CV fractions until proteins concentrations dropped below 0.05 mg/ml to ensure removal of all excess 14-3-3. 14-3-3ζ/ERα complex was then eluted using 2 to 3 CV Elution Buffer (20 mM Tris, pH 7.5, 300 mM NaCl, 500 mM imidazole, 2% (v/v) glycerol, 2 mM βME). The 14-3-3ζ-strep/His-SUMO-ERα complex was subsequently incubated with SUMO hydrolase (1:100) to remove the His-SUMO tag from ERα during overnight dialysis at 4 °C (20 mM Tris, pH 7.5, 150 mM NaCl, 10 mM MgCl_2_, 5% (v/v) glycerol, 2 mM βME). SUMO-cleaved 14-3-3ζ-strep/ERα complex was loaded on a 5 ml Streptavidin column (Strep-TactinXT 4Flow cartridge, IBA) at 3 ml/min. Columns were washed with Buffer W (100 mM Tris pH 8.0, 150 mM NaCl, 1 mM EDTA) collecting 2 CV fractions until protein concentrations dropped below 0.05 mg/ml to ensure removal of all excess ERα. 14-3-3ζ/ERα complex was eluted using 8 CV Buffer BXT (100 mM Tris pH 8.0, 150 mM NaCl, 1 mM EDTA, 50 mM biotin). Finally, the protein complex was purified by SEC (Superdex 75pg Hiload 16/600) at 1 ml/min and 4 °C using SEC buffer (20 mM Tris pH 7.5, 150 mM NaCl, 10 mM MgCl_2_, 0.5 mM TCEP). Protein-containing fractions were combined and concentrated to >10 mg/ml, aliquoted and stored at −80 °C. Correct mass and purity of the proteins was confirmed using SDS-PAGE and Q-ToF LC-MS analysis.

### Analytical SEC

Protein samples were diluted in 20 mM Tris pH 7.5, 150 mM NaCl, 10 mM MgCl_2_, and 0.5 mM TCEP to a final concentration of 5 to 10 μM. All analytical SEC experiments were performed on an Agilent 1260 bio-inert HPLC in combination with a Superdex200 increase 3.2/300 column at a flow rate of 0.075 ml/min and 20 mM Tris pH 7.5, 150 mM NaCl, 10 mM MgCl_2_, and 0.5 mM TCEP as running buffer. Peak detection was performed by absorbance measurements at 280 nM.

### Sedimentation-velocity AUC

Protein samples were dialyzed into 20 mM HEPES pH 7.5, 150 mM NaCl, 10 mM MgCl_2_, and 0.5 mM TCEP before all AUC measurements to obtain the best buffer match between the blank and the sample. Protein samples were diluted to their final concentrations in dialysis buffer and ligands were added where described. Samples were placed into double sector titanium centerpieces with 12-mm optical path length. SV-AUC experiments were performed using a ProteomLabTM XL-I analytical ultracentrifuge (Beckman Coulter) at 20 °C and at 43.000 to 45.000 rev./min rotor speed (An-50 Ti rotor, Beckman Coulter). All sedimentation profiles were collected by absorbance measurements at 280 nm. The calculated distributions were integrated to establish the weight-average sedimentation coefficients corrected to 20 °C and to the density of water (*s*_*w(20,w)*_).

### QToF-MS quantification

Dilution series of 14-3-3ζ-strep and ERα-pT594-strep were prepared in MQ (0.1% FA) to final concentrations of 0.025, 0.020, 0.015, 0.010, and 0.005 mg/ml. Furthermore, a 500×, 750× and 1000× dilution of the ERα/14-3-3ζ protein complex were prepared. The final samples (∼100 μl) were transferred to a 200 μl LC-MS vial. UPLC-QToF-MS analysis was performed on a Waters (Milford, MA, USA) Acquity I-Class UPLC system coupled to a Waters Xevo G2-XS quadrupole time-of-flight (QToF) mass spectrometer. The devices were controlled by MassLynx Software (version 4.2, Waters). Full scan in positive electrospray ionization (ESI+) mode was used as MS acquisition mode with an acquisition range from 150 to 2000 m/z. A 3 μm, 100 × 2.0 mm Polaris 3 C8-A column (Agilent, Middelburg, the Netherlands) was placed inside a column oven at 40 °C and used for chromatographic separation. Flowrate was set at 0.3 ml/min, and a gradient of water containing 0.1% (v/v) formic acid (A) and acetonitrile containing 0.1% (v/v) formic acid (B) was set as follows (all displayed as % v/v): 0.0 to 2.0 min (30% to 39% B), 2.0 to 5.0 min (39% B), 5.0 to 7.5 min (39% to 60% B), 7.5 to 8.0 min (60% B), 8.0 to 8.1 min (60% to 30% B) 8.1 to 10.0 min (30% B). Sample injection volume 2 μl. Mass Spectrometry settings were set as follows: capillary voltage: 0.80 kV, cone voltage: 40 V, source offset: 80 V, source temperature: 120 °C, desolvation temperature: 450 °C, cone gas: 10 l/h desolvation gas: 1000 l/h.

Data were analyzed using MassLynx software. Chromatograms were background subtracted (polynomial order 1, below curve 40%, tolerance 0.010, flatten edges). The area under the peak was determined using integration in the MassLynx software with a relative area threshold of 10. The obtained area under the curve was then plotted against the protein concentration, after which a linear regression was determined between the five data points. Using the equation of the linear regression, the concentration of 14-3-3 and ERa was determined in each protein complex sample.

To perform mass analysis of the individual peaks deconvolution was performed on m/z spectra of each individual peak. After visual inspection of the m/z spectrum, the spectrum was zoomed to the five most abundant peaks from which the mass spectrum was determined using MaxEnt1 (mass ranges 27–30 kDa or 32–36 kDa; resolution 0.10 Da/channel, Simulated Isotope Pattern with Spectrometer Blur width 0.32–0.38 Da, minimum intensity ratios left 33%, right 33%, iterate to converge). Mass spectra were centered and errors of the deconvolution process were determined.

### HDX - peptide mapping

100 pmol of 14-3-3ζ-strep or ERα(PKA)-strep was mixed in 1:1 (v/v) ratio with 1 M glycine at pH 2.3 and injected on a mixed Pepsin/Nepenthesin-2 acidic protease column. Generated peptides were trapped and desalted by a Micro trap column (Luna Omega 5 um Polar C18 100 Å Micro Trap 20 × 0.3 mm) for 3 min at a flow rate 200 μl min^−1^ using isocratic pump delivering 0.4% formic acid in water. Both protease column and trap column were placed in an icebox. After 3 min, peptides were separated on a C18 reversed-phase column (Luna Omega 1.6 μm Polar C18 100 Å, 100 × 1.0 mm) with a linear gradient 5 to 35% B in 26 min, where solvent A was 2% acetonitrile/0.4% formic acid in water and solvent B 95% acetonitrile/5% water/0.4% formic acid. The analytical column was placed in an icebox. TimsToF Pro mass spectrometer (Bruker Daltonics) operating in positive MS/MS mode was used for the detection of peptides. Data were processed by DataAnalysis 5.3 software (Bruker Daltonics). MASCOT search engine was used for the identification of peptides using a database containing the sequence of 14-3-3ζ or ERα.

### HDX

All proteins were dialyzed and diluted into 20 mM Hepes, 150 mM NaCl, 10 mM MgCl2, and 0.5 mM TCEP pH7.5 to a final concentration of 20 μM. E2 or DMSO was added to a final concentration of 150 μM. Hydrogen deuterium exchange was initiated by 10-fold dilution of the proteins under different conditions in a deuterated buffer. Fifty microliter aliquots (100 pmol) were taken after 20 s, 2 min, 20 min and 2 h of incubation in deuterated buffer, quenched by 50 μl of 1 M glycine, pH 2.3 and snap frozen in liquid nitrogen. Aliquots were quickly thawed and analyzed using the same system as described above. Peptides were separated by linear gradient 10 to 30% B in 18 min. Mass spectrometer was operated in positive MS mode. Spectra of partially deuterated peptides were processed by Data Analysis 5.3 (Bruker Daltonics) and by in-house program DeutEx.

### Native PAGE

Samples were prepared in 20 mM Tris pH 7.5, 150 mM NaCl, 10 mM MgCl_2_, and 0.5 mM TCEP with protein at a final concentration of 2.5 to 5 μM. Ligands were added at a final concentration of 100 μM. All samples were 1:1 diluted into native PAGE loading dye (62.5 Tris pH 7.1, 75 mM NaCl, 5 mM MgCl_2_, 20% glycerol, 0.01% bromphenolblue). after which 12 μl of each sample was loaded on a 4 to 20% Mini-PROTEAN TGX Precast Protein Gel (Bio-Rad). Gels ran at 130 V for 2.5 h at 4 °C in running buffer (25 mM Tris, 192 mM Glycine, pH 8.3). Gels were washed in MilliQ (20 min), stained with Coomassie Brilliant Blue G-250 (Bio-Rad), and destained in MilliQ until bands were clearly visible. Gels were imaged and analyzed with ImageJ.

### Differential scanning fluorimetry

Proteins were diluted (in 20 mM Hepes pH 7.5, 150 mM NaCl, 10 mM MgCl_2_, 500 μM TCEP) to obtain 40 μl samples containing 5 μM 14-3-3ζ-strep, 5 μM ERα-strep or 10 μM ERα/14-3-3ζ complex, with either 1% DMSO (negative control) or 100 μM ligand (E2, 4-OHT, FC-A). All samples additionally contained 10x ProteoOrange dye (Lumiprobe, 5000x stock in DMSO) and were heated from 35 to 79 °C at a rate of 0.3 °C per 15 s in a CFX96 Touch Real-Time PCR Detection System (Bio-Rad). Fluorescence intensity was determined using excitation 575/30 nm and emission 630/40 nm filters. Based on these melting curves, the (negative) first derivative melting curve was obtained, from which the melting temperature T_M_ could be determined. Reported T_M_ values were obtained from three independent experiments from which the average and standard deviations were determined using excel.

### Fluorescence anisotropy

All FA dilution series were prepared in polystyrene (non-binding) low-volume Corning Black Round Bottom 384-well plates (Corning 4514 or 4511). FA measurements were performed directly after plate preparation, using a Tecan Infinite F500 plate reader at room temperature (l_ex_: 485 ± 20 nm; l_em_: 535 ± 25 nm; mirror: Dichroic 510; flashes: 20; integration time: 50 ms; settle time: 0 ms; gain: 60; and Z-position: calculated from well). Wells containing only fluorescein-labeled peptide were used to set as G-factor at 35 mP. All data were analyzed using GraphPad Prism (7.00) for Windows and fitted using a four-parameter logistic model (4PL) to determine apparent binding affinities (K_D_^app^). All results are based on two independent experiments from which the average and standard deviations were calculated to obtain the final values.

### FA - SCR1 cofactor peptide binding studies

ERα(PKA)-strep, ERα(PKA)-pT594-strep, and ERα/14-3-3ζ protein complex (for WT ERα and Y537S mutant) were titrated in a 2-fold dilution series (starting at 40 μM ERα) to 20 nM of fluorescein-labeled SRC1 peptide in 20 mM HEPES pH 7.5, 150 mM NaCl, 10 mM MgCl_2_, 0.1% (v/v) Tween20, 0.1% (w/v) BSA. This is done in the presence of DMSO (negative control), 100 μM E2 or 100 μM 4-OHT.

### FA - ERα peptide mimic binding studies

14-3-3ζ was titrated in a 2-fold dilution series (starting at 300 μM 14-3-3ζ) to 2 nM of fluorescein-labeled peptide (WT-ERα, ERα(PKA), ERα(PKA)-strep) in 20 mM HEPES pH 7.5, 150 mM NaCl, 10 mM MgCl_2_, 0.1% (v/v) Tween20, 0.1% (w/v) BSA. This is done in the presence of a 2-fold dilution series of 3′de-Ac FC-A (0.015–250 μM); each well contained a final concentration of 0.25% DMSO.

### X-ray crystallography data collection and refinement

N-acetylated ERα(PKA)-pT594 and ERα(PKA)-pT594-strep peptides were mixed with 10 mg/ml 14-3-3σΔC protein (truncated after T231 to reduce flexibility) in complexation buffer (20 mM HEPES pH 7.5, 100 mM NaCl, 10 mM MgCl_2_ and 20 μM TCEP) using a final molar stoichiometry of 1:2 or 1:4 protein to peptide. These complexes were used to set up sitting-drop crystallization plates were set up in which each of the four complexation mixtures were combined with 24 crystallization buffers, optimized for 14-3-3σ crystallization (0.095 M HEPES (pH7.1, 7.3, 7.5, 7.7), 0.19 M CaCl_2_, 24 to 29 % (v/v) PEG 400 and 5% (v/v) glycerol). For each combination, a 1:1 mix (both 250 nl) of complexation mixture and crystallization buffer was prepared for crystal growth. Crystals grew within 10 to 14 days at 4 °C. Co-crystals of 14-3-3ζ with ERα peptides (ERα(PKA)-pT594 and ERα(PKA)-pT594-strep) were soaked with 10 mM 3′ deAc-FC-A in crystallization buffer and incubated for 3 days at 4 °C.

Both soaked and non-soaked crystals were fished and flash-frozen in liquid nitrogen. X-ray diffraction data were collected at the p11 beamline of PETRA III facility at DESY (Hamburg, Germany) with the following settings: 1440 image, 0.25°/image, 100% transmission, and 0.1 s exposure time. Initial data processing was performed at DESY using XDS after which pre-processed data was taken to further scaling steps, molecular replacement, and refinement.

Data were processed using the CCP4i2 suite (version 7.1.18). XDS-preprocessed data were scaled using AIMLESS. The data were phased with MolRep, using protein data bank (PDB) entry 4JC3 as a template. A three-dimensional structure of 3′ dAc-FC-A was generated using AceDRG, which was thereafter built in the electron density based on visual inspection Fo-Fc and 2Fo-Fc electron density map. Sequential model building (based on visual inspection Fo-Fc and 2Fo-Fc electron density map) and refinement were performed with Coot and REFMAC, respectively. Finally, alternating cycles of model improvement (based on isotropic b-factors and the standard set of stereo-chemical restraints: covalent bonds, angels, dihedrals, planarities, chiralities, non-bonded) and refinements were performed using Coot and phenix.refine from the Phenix software suite (version 1.20.1-4487). Pymol (version 2.2.3) was used to make the figures in the manuscript. All structures were deposited in the protein data bank (PDB) and obtained IDs: 8C40, 8C42, 8C3Z and 8C43. See Table S1 for x-ray crystallography data statistics.

## Data availability

Crystal structures described in this manuscript have been deposited to the PDB. They have the following PDB codes: 8C40, 8C42, 8C3Z, and 8C43.

## Supporting information

This article contains supporting information.

## Conflict of interest

The authors declare the following competing interest(s): L. B. and C. O. are scientific co-founders of Ambagon Therapeutics. C. O. and E. S. are employees of Ambagon Therapeutics.
