# Supplementary material for: Molecular basis and dual ligand regulation of tetrameric estrogen receptor α/14-3-3ζ protein complex
Source: J Biol Chem. 2023 May 22;299(7):104855. doi: 10.1016/j.jbc.2023.104855 (PMC10302166; doi:10.1016/j.jbc.2023.104855)
Supplement: Supporting information [file mmc1.pdf]

Molecular basis and dual ligand regulation of tetrameric Estrogen Receptor α/14-3-3ζ protein complex

Bente A. Somsen[1], Eline Sijbesma[1], Seppe Leysen[1], Karolina Honzejkova[2], Emira J. Visser[1], Peter J. Cossar[1], Tomáš Obšil[2], Luc Brunsveld[1*], and Christian Ottmann[1*]

From the [1] Laboratory of Chemical Biology, Department of Biomedical Engineering and Institute for Complex Molecular Systems, Eindhoven University of Technology, PO Box 513, 5600 MB Eindhoven, The Netherlands; and [2]Department of Physical and Macromolecular Chemistry, Faculty of Science, Charles University, Prague, Czech Republic.
.

Supporting Information

Content:

Supplementary Figures and Tables……………………………………………………………………..………..2-28
Protein constructs……………………………………..……………………………………………………………… 29

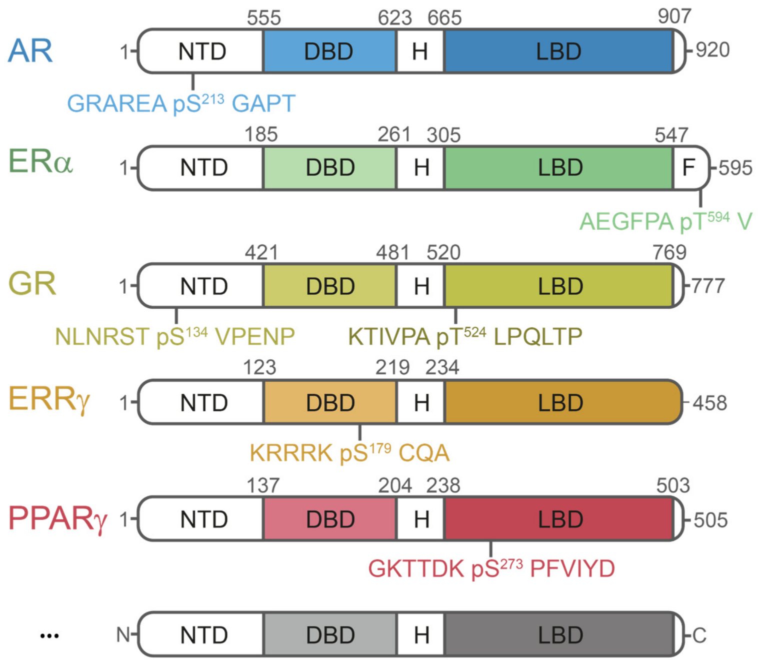

Figure S1 | 14-3-3/NR PPI stabilizers. Schematic representation of NR domain organization of five 14-3-3 binding NRs. For each NR, the 14-3-3 binding sequence is shown as phosphopeptide sequence.

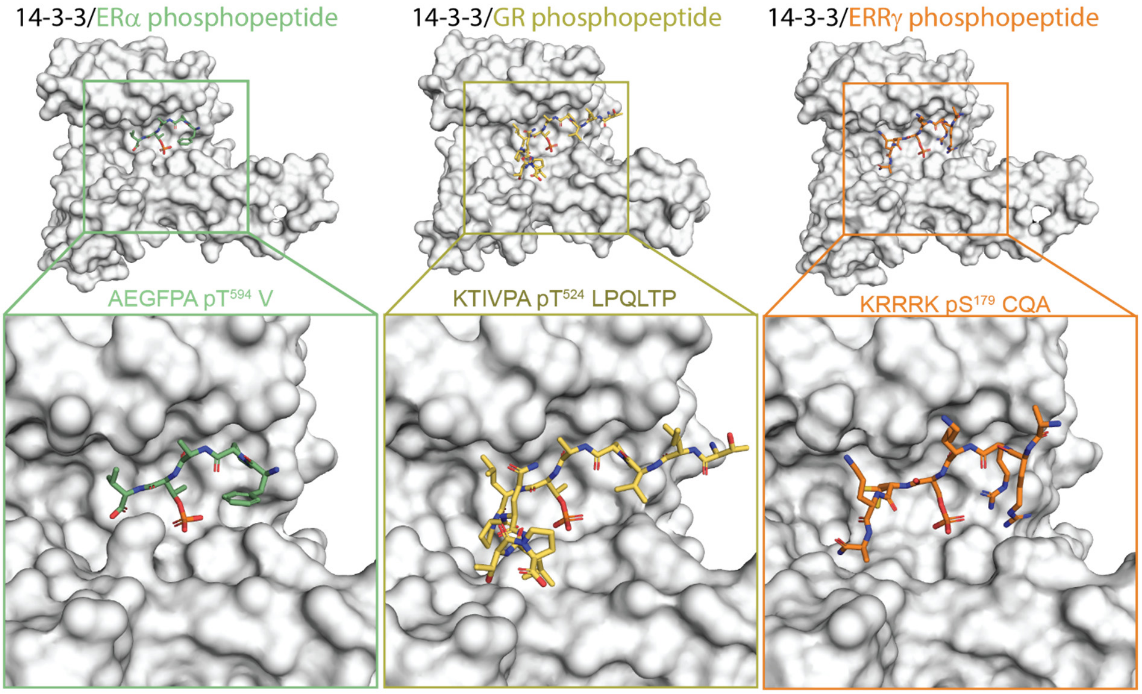

Figure S2 | 14-3-3/NR peptide interactions. Crystal structures of 14-3-3σ (with surface) in complex with ERα F-domain, ERRγ DBD, or GR LBD phosphopeptides (green/yellow/orange sticks). PDB: 4JC3, 6Y1D & 6Y08.

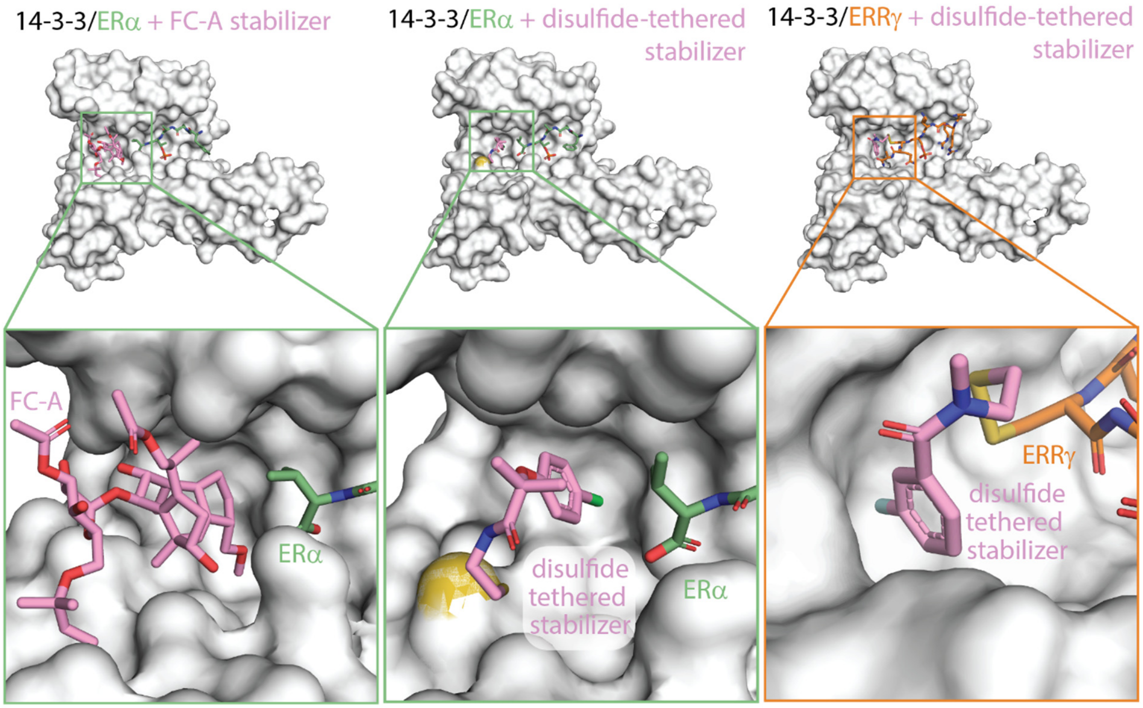

Figure S3 | 14-3-3/NR PPI stabilizers. Crystal structures of 14-3-3σ (with surface) in complex with ERα F-domain or ERRγ DBD phosphopeptides (green/orange sticks) with PPI stabilizing small molecules (FC-A and disulfide tethered small molecules, pink sticks). PDB: 4JDD, 6HMT & 6Y3W.

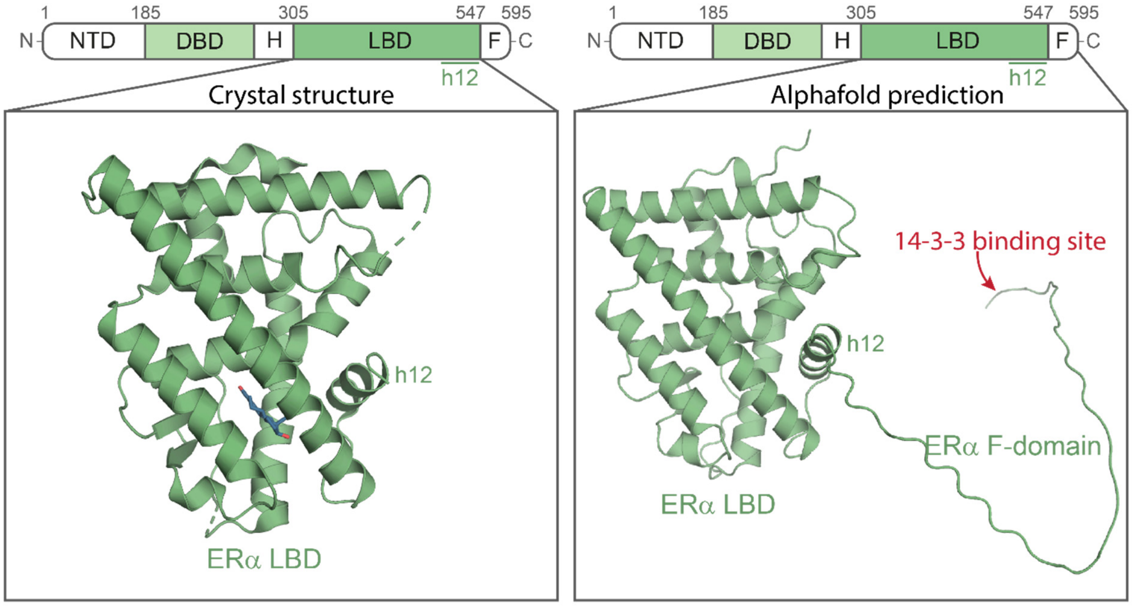

Figure S4 | ERα LDB-F structures. (left) ERα domain structure and the crystal structure of the ERα ligand binding domain (LBD) (green cartoon) bound by E2 (blue sticks). PDB: 5WGD. (right) ERα domain structure and the alphafold predicted structure of the ERα ligand binding domain (LBD) and F-domain (green cartoon).[71,72]

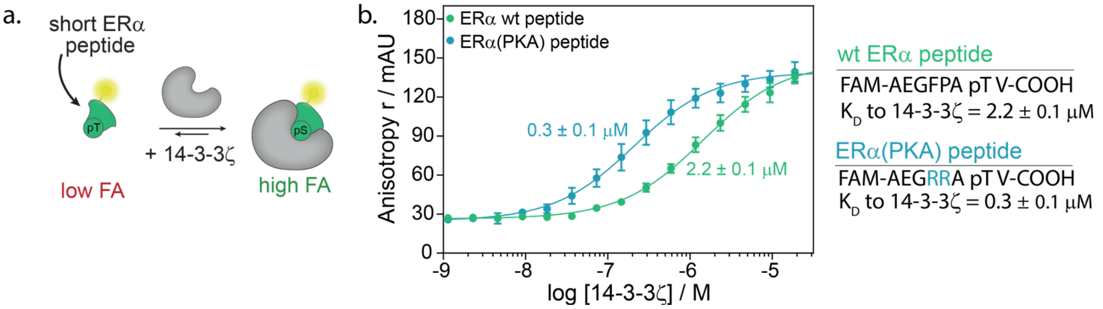

Figure S5 | Effect PKA mutations on 14-3-3 binding. (a) Schematic representation of Fluorescence Anisotropy (FA) assay where 14-3-3z is titrated to a short fluorescently labelled peptide representing ERa. Binding of 14-3-3z to ERa slows down in its tumbling rate, thereby increasing FA levels. (b) Fluorescence anisotropy assay of 14-3-3ζ titration to 2 nM fluorescein labelled ERα wt peptide and PKA-responsive ERα peptide. Data shown is an average and standard deviation of two independent experiments. Peptide sequence of wildtype ERα F-domain peptide and PKA-responsive ERα F-domain peptide are shown at the right.

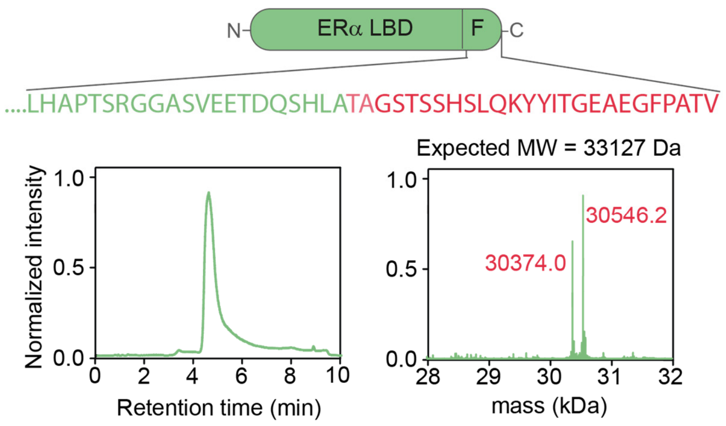

Figure S6 | Truncation ERα LDB-F protein. Results of ERα LBD-F protein bacterial expression and purification using QTof-MS. High-resolution MS chromatogram and mass spectrum of purified protein show two different masses corresponding truncation of ERα at residues 570 and 572 of ERα F-domain.

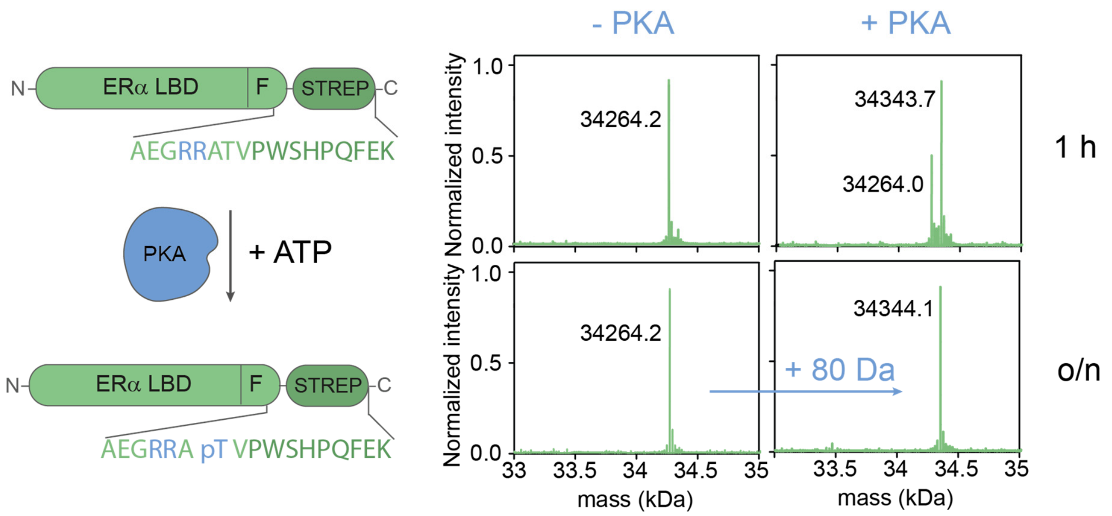

Figure S7 | Phosphorylation ERα(PKA)-strep. In vitro phosphorylation assay of ERα(PKA)-strep with PKA. QToF-MS was used to follow the protein phosphorylation. Results show time dependent phosphorylation of ERα(PKA)-strep in presence of PKA. Expected mass non-phosphorylated: 34264.6Da. Expected mass non-phosphorylated: 34344.6 Da.

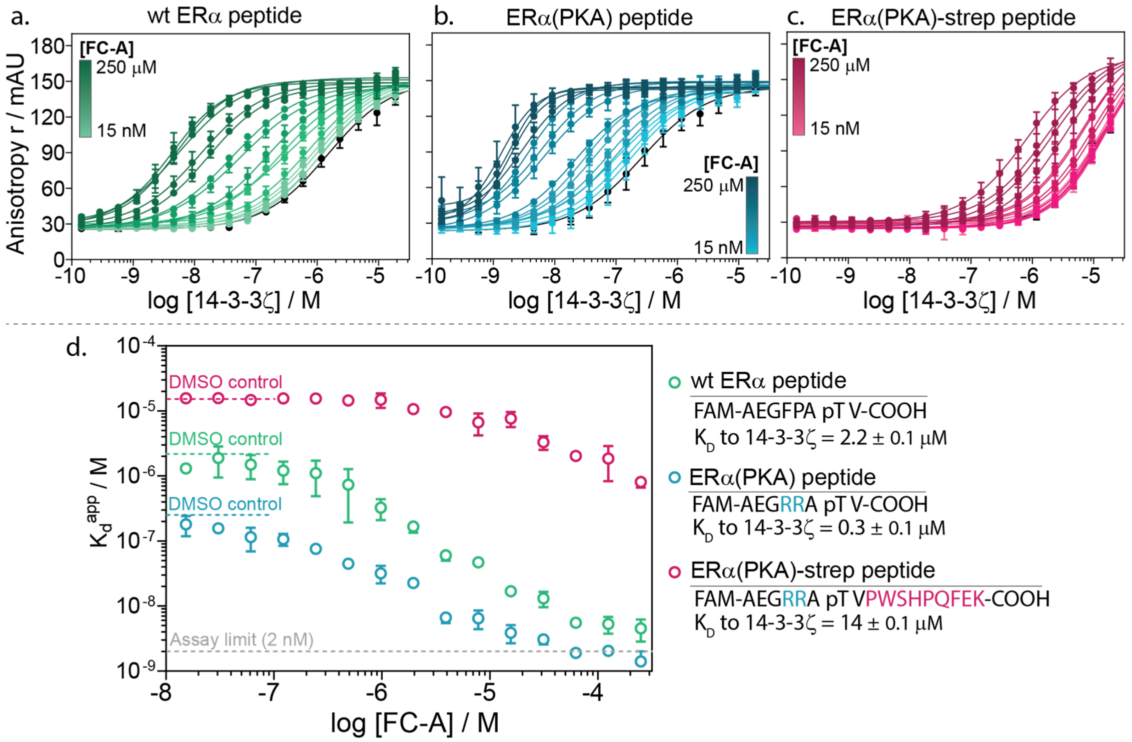

Figure S8 | 2D-FA of 14-3-3/ERα/FC-A. (a-c) Fluorescence anisotropy assay of 14-3-3ζ titration to 2 nM fluorescein labelled ERα wt, ERα(PKA) and ERα(PKA)-strep phosphopeptides in presence of a 2-fold dilution series of 3'deAc-FC-A (15 nM to 250 μM) showing enhance binding affinity between 14-3-3ζ and ERα upon addition of 3'deAc-FC-A. (d) Concentration of 3'deAc-FC-A plotted against the apparent K_D between 14-3-3ζ and the three ERα phosphopeptides showing a decrease in K_D upon addition of 3'deAc-FC-A. Data shown is an average and standard deviation of two independent experiments.

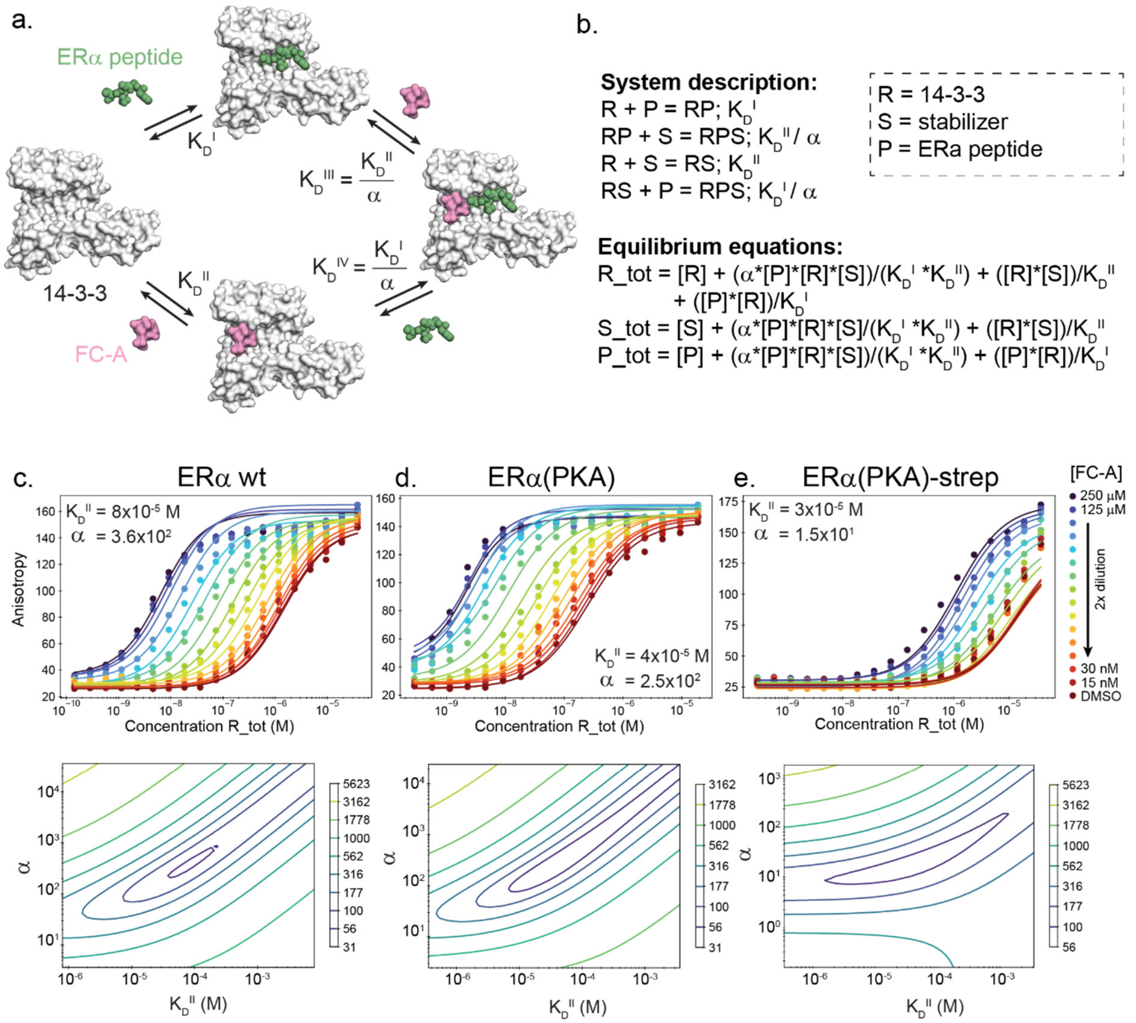

Figure S9 | Cooperativity analysis. (a) Cooperativity square representation of the 14-3-3/ERα/FC-A complex formation including binding affinities. The interaction is stabilized by factor alpha (α), reducing the apparent affinity of 14-3-3 to either of the components when pre-bound to the other component. (b) System description given to model to determine our ternary complex formation system and the determined equilibrium equations as generated by the model. (c-e) Experimental fluorescence anisotropy data of 14-3-3ζ titration to FITC-labelled ERα peptides in presence of several concentrations 3'deAc-FC-A with corresponding plots by thermodynamic model. Calculated K_D^{II} and α factor are given for each peptide. Als for each peptide, an error-landscape plot centered on the determined K_D^{II} and α factors is provided.

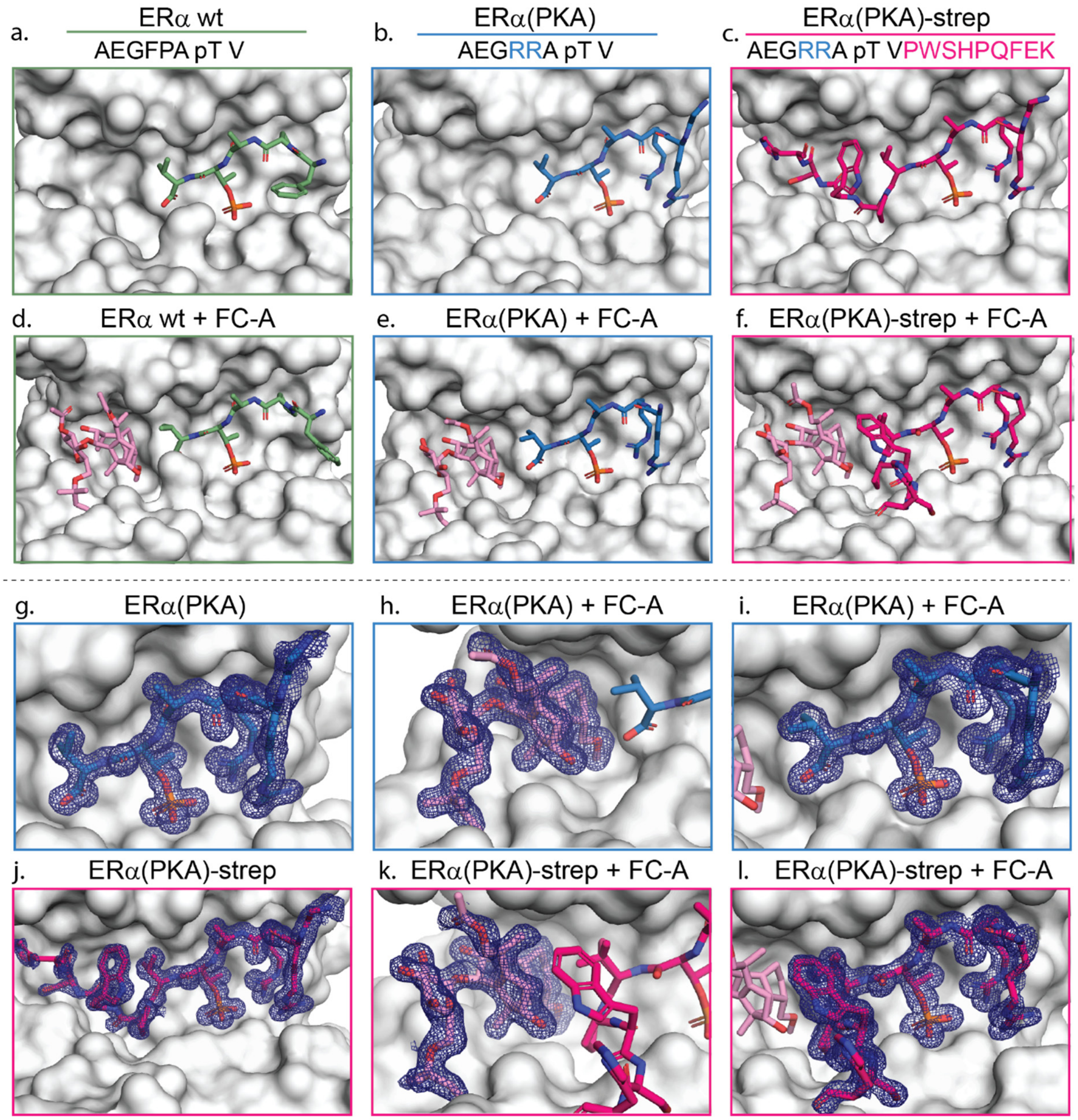

Figure S10 | 14-3-3/NR PPI stabilizers. (a-c) Crystal structures of 14-3-3σ (with surface) in complex with wt ERα, ERα(PKA), and ERα(PKA)-strep phosphopeptides (coloured sticks). PDB: 4JC3, 8C40 & 8C3Z. (d-f) Crystal structures of 14-3-3σ (with surface) in complex with wt ERα, ERα(PKA), and ERα(PKA)-strep phosphopeptides (coloured sticks) in presence of FC-A or 3'deAc-FC-A (pink sticks). PDB: 4JDD, 8C42 & 8C43. (g-l) Crystal structures of 14-3-3σ (with surface) in complex with ERα(PKA) or ERα(PKA)-strep phosphopeptides (coloured sticks) in presence and absence of 3'deAc-FC-A (pink sticks). The 2Fo – Fc electron density map (blue mesh) is contoured at 1σ. PDB: 8C40, 8C42, 8C3Z & 8C43.

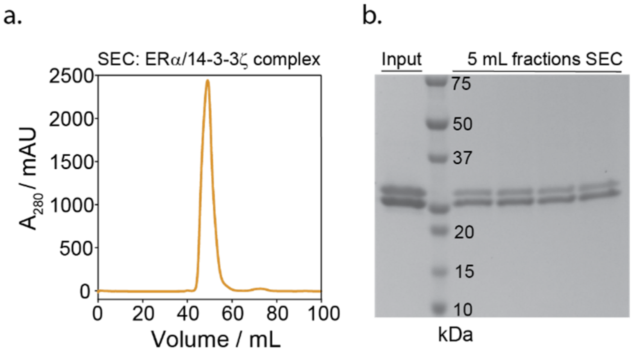

Figure S11 | SEC purification of ERα/14-3-3ζ complex. (a) Typical size exclusion chromatograph (SEC) run of ERα/14-3-3ζ protein complex during protein purification. (b) Coomassie stained SDS-PAGE gel from SEC run with the sample as input and the 5 mL fractions that contained the ERα/14-3-3ζ protein complex.

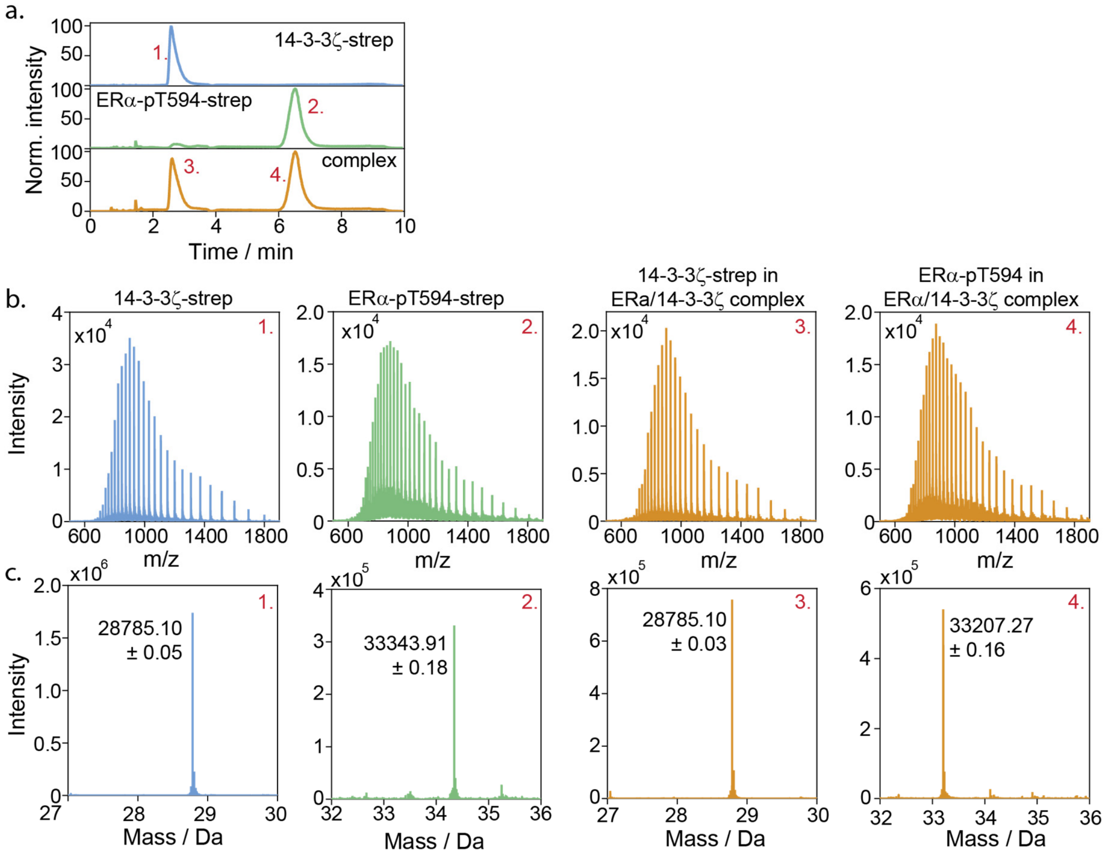

Figure S12 | QToF-MS of 14-3-3ζ and ERα proteins. (a) Chromatogram of high-resolution MS of 14-3-3ζ-strep (blue), ERα-pT594-strep (green) and the ERα/14-3-3ζ protein complex (orange). (b). m/z spectra of 14-3-3ζ-strep (blue), ERα-pT594-strep (green) and 14-3-3ζ-strep (orange) and ERα-pT594 (orange) within the ERα/14-3-3ζ protein complex. m/z spectra were obtained from the peaks in figure S12a. (c) Mass spectra of 14-3-3ζ-strep (blue, expected mass: 28785.1 Da), ERα-pT594-strep (green expected mass: 33344.6 Da) and 14-3-3ζ-strep (orange, expected mass: 28785.1 Da) and ERα-pT594 (orange, expected mass: 33207.3 Da) within the ERα/14-3-3ζ protein complex. Mass spectra are obtained from deconvolution of the m/z spectra in figure S12b.

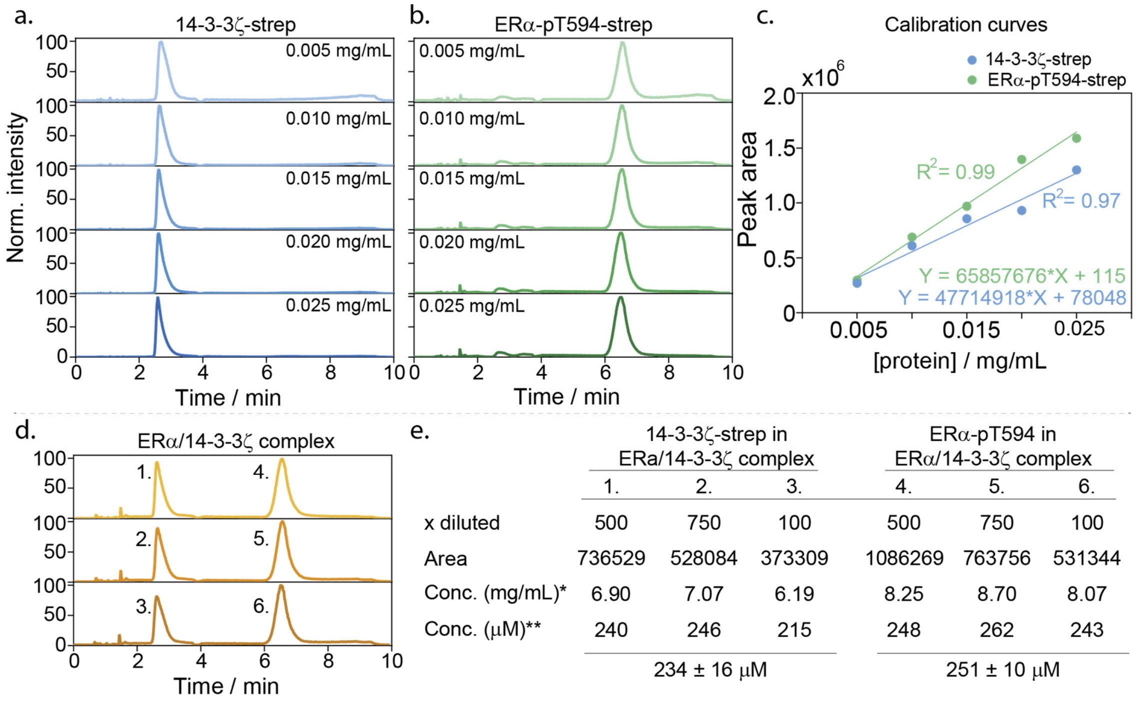

Figure S13 | QToF-MS quantification 14-3-3ζ and ERα proteins. (a) LC chromatogram of a dilution series of 14-3-3ζ-strep (0.005-0.025 mg/mL) (b). LC chromatogram of a dilution series of ERα-pT594-strep (0.005-0.025 mg/mL). (c) Calibration curves of 14-3-3ζ-strep and ERα-pT594-strep which correlates protein concentration to area under the curve in the chromatograms in a and b. A linear regression is plotted through these points giving the provided equations and R^2 values. (d)) LC chromatogram of three samples of ERα/14-3-3ζ protein complex. (e) Calculations of 14-3-3ζ-strep and ERα-pT594 concentrations based on the area under the curve and the calibration curve. *Concentration in mg/mL is determined using the linear equation of each protein (Y = area under the curve) times the dilution of the sample. ** Concentration in mM is determined using concentration in mg/mL divided by the molecular weight.

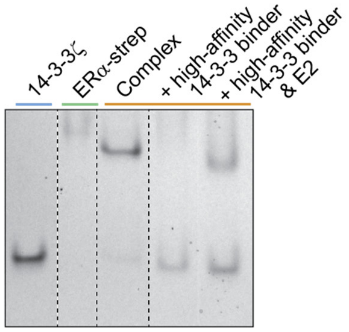

Figure S14 | Native page. Native page gel of 14-3-3ζ (blue), ERα-strep (green) and the ERα/14-3-3ζ complex (orange). A high-affinity 14-3-3 binder (14-3-3.6)[62] was added to the complex which shows disappearance of the complex band and appearance of the 14-3-3 band. Addition of E2, which increases stability of ERα protein, to this mixture also shows presence of the ERα band.

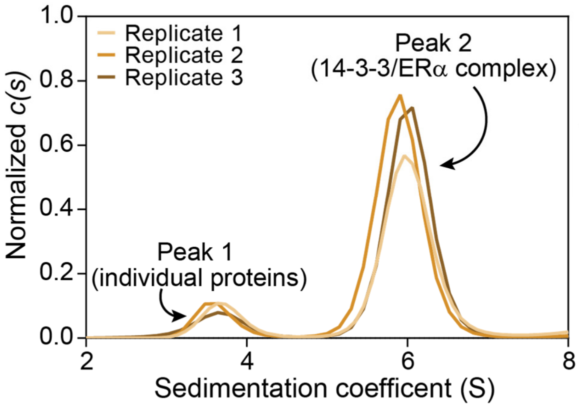

	1	2	3
[14-3-3ζ dimer]* (μM)	2.5	2.5	2.5
[ERα dimer]* (μM)	2.5	2.5	2.5
Peak 1 (individual)	4.6	4.5	4.5
% individual (area)	11.9%	10.1%	10.0%
Peak 2 (complex)	6.0	5.9	6.0
% complex (area)	88.1%	89.9%	90.0%
[complex]** (μM)	2.20	2.25	2.25
K_D*** (nM)	40	28	28

Figure S15 | K_D determination. Three replicates of *c(s)* distributions of three replicates of ERα/14-3-3ζ protein complex indicating one peak for individual proteins (dimeric) and a second peak of the protein complex. Based on the area under these peaks and the concentration of 14-3-3ζ and ERα dimers (*input in assay, assuming 100% dimer formation) in the sample, the concentration of complex in the sample was determined (** [14-3-3ζ] x % in complex). Based on the amount of complex, and the concentration of the individual protein, the K_D was determined (*** K_D = (([14-3-3ζ dimer]-[complex])([ERα dimer]-[complex]))/[complex]).

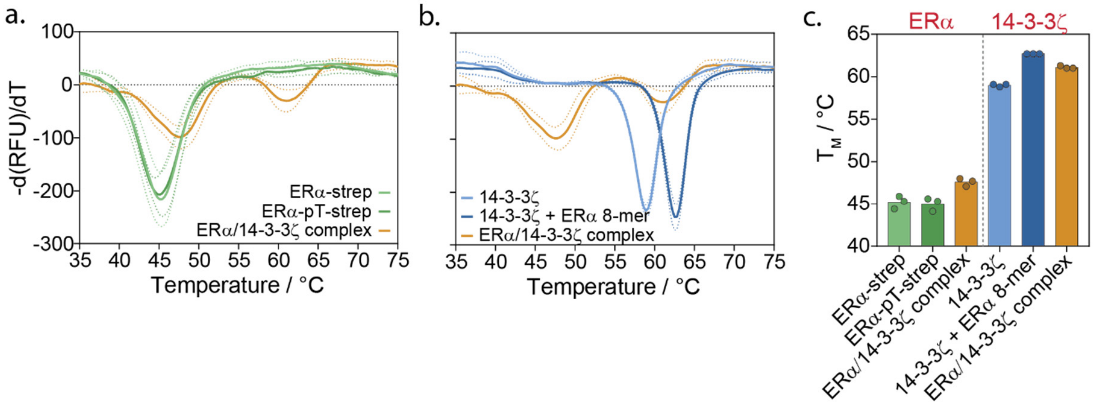

Figure S16 | DSF of 14-3-3ζ/ERα protein complex. (a) Differential melting curve of 5 μM ERα-strep (green), 5 μM ERα-pT594-strep (green) and 10 μM ERα/14-3-3ζ complex (orange). (b) Differential melting curve of 5 μM 14-3-3ζ (blue), 5 μM 14-3-3ζ + 50 μM ERα 8-mer phosphopeptide (bleu) and 10 μM ERα/14-3-3ζ complex (orange). (c) Melting temperatures (T_M) of ERα and 14-3-3ζ individually and when present in complex.

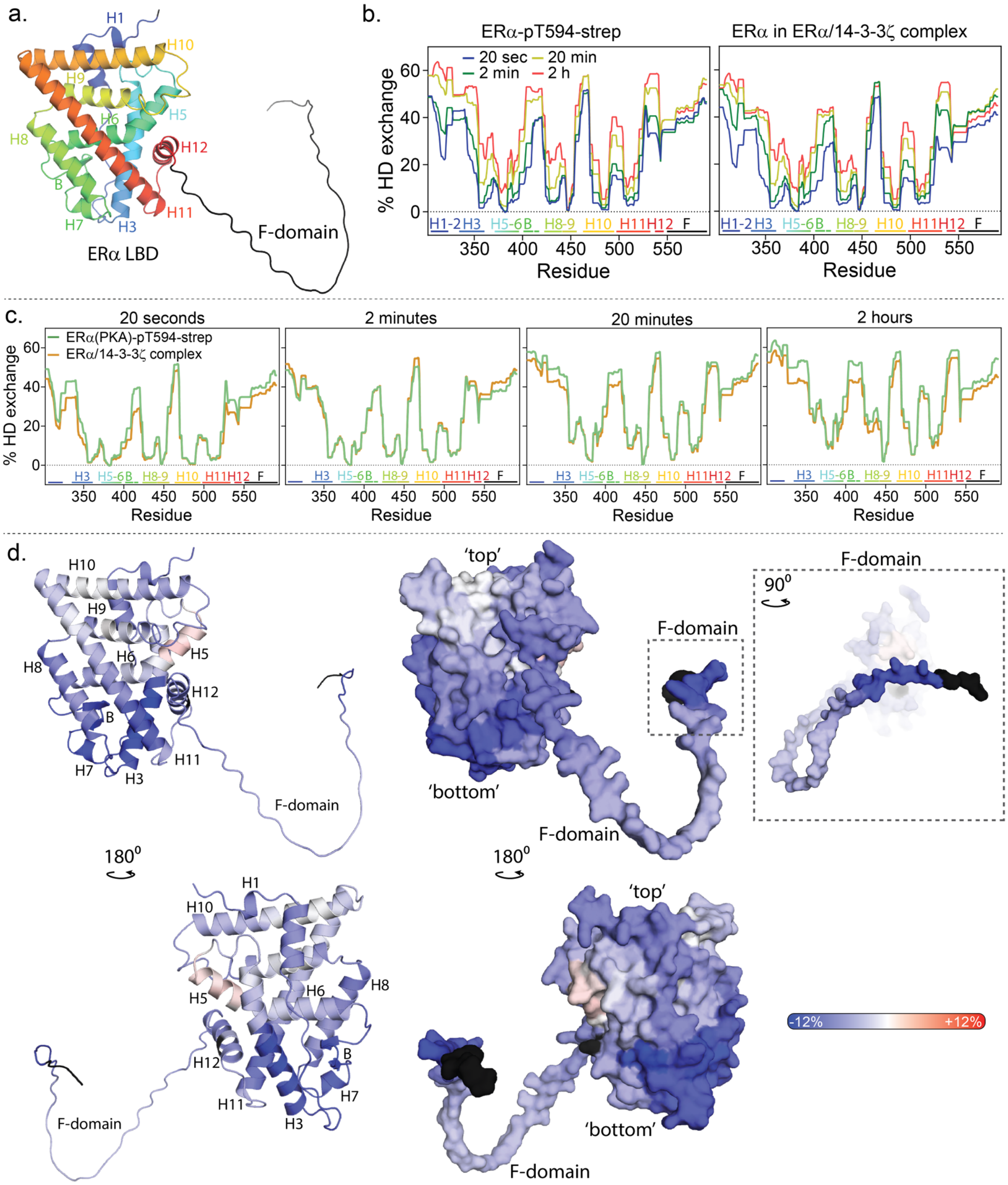

Figure S17 | HDX of ERα. (a) Predicted Alphafold structure of ERα ligand binding domain (LBD) and F-domain in rainbow colors with the N-terminal side colored in blue and the C-terminal side in red. Furthermore each alpha helix (H) and the beta sheets (B) are labelled with corresponding colors. (b) HDX-MS exchange profile of ERα-pT594-strep and ERα in the ERα/14-3-3ζ protein complex after 20 sec (blue), 2 min (green), 20 min (yellow) and 2 h (red) of HDX. Plots show the amount of deuteration of each residue in the ERα protein. (c) HDX-MS exchange profile of ERα-pT594-strep (green) and ERα in the ERα/14-3-3ζ protein complex (orange) after 20 sec, 2 min, 20 min and 2 h. Plots show the effect of 14-3-3ζ binding for each residue at various time points. (d) HDX difference profile of ERα/14-3-3ζ complex minus ERα-pT594-strep after 2h of HDX, which displays the effect of 14-3-3ζ binding on deuteration of ERα-pT594. Shielding effect (less deuterium exchange) is shown in blue, deshielding effects (increase in deuterium exchange) in red, unavailable data is shown in black. Results are displayed on the predicted Alphafold structure of the ERα LBD and F domains.

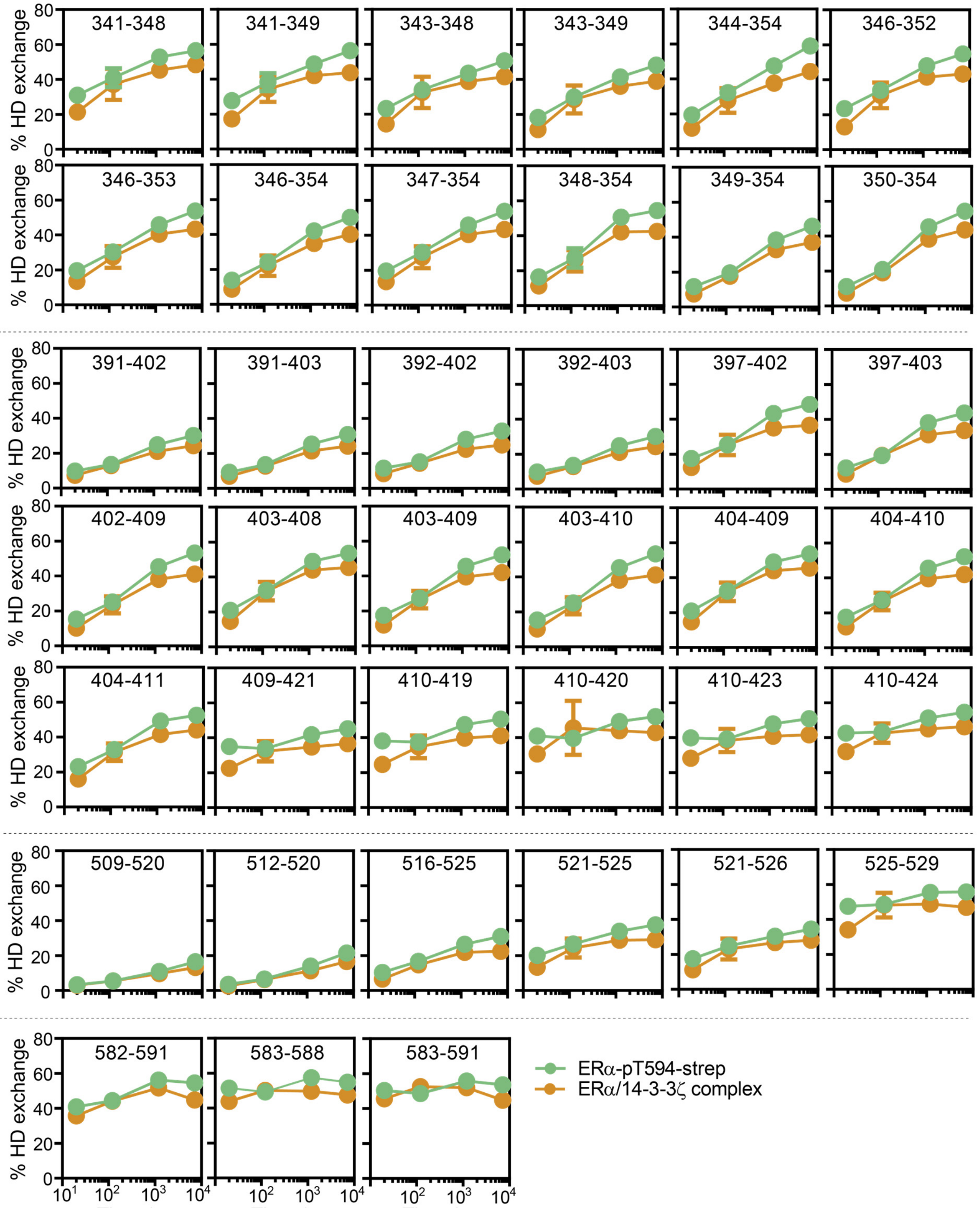

Figure S18 | HDX peptides of ERα. Hydrogen-deuterium exchange levels of individual peptides within ERα after 20 seconds, 2 minutes, 20 minutes and 2 hours. Data is shown for ERα-pT594-strep (green) and ERα-pT594 in the ERα/14-3-3ζ protein complex (orange). Peptides are represented for regions that showed significant changes in HDX profiles after 2h incubation. Here shown, residues 341-354 in helix 3, residues 391-424 in the beta sheets and helix 7, residues 509-529 in helix 11 and residues 583-591 in the F-domain.

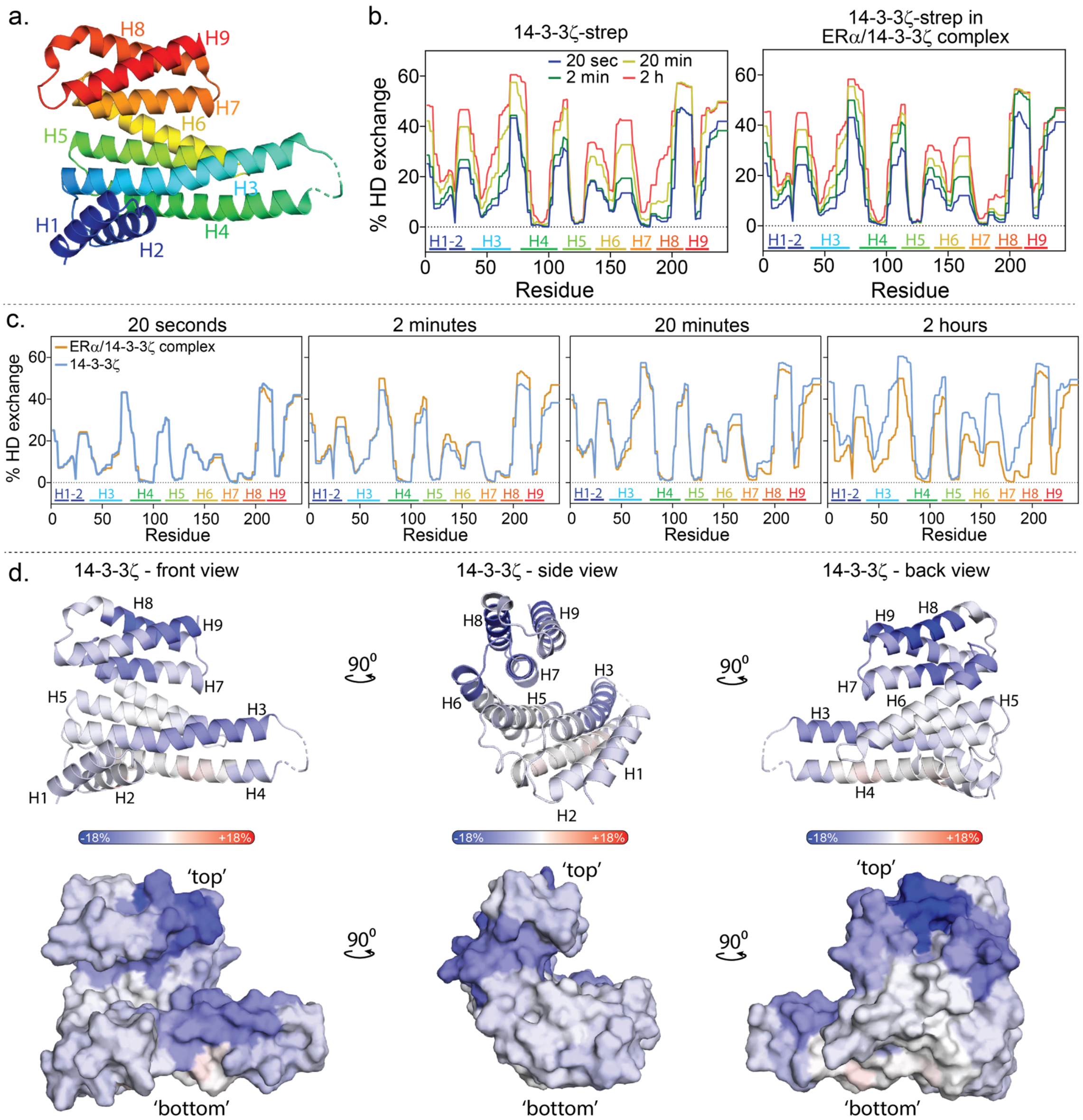

Figure S19 | HDX of 14-3-3ζ. (a) Crystal structure of 14-3-3ζ in rainbow colors with the N-terminal side colored in blue and the C-terminal side in red. Furthermore each alpha helix (H) is labelled with corresponding colors. PDB: 6F09 (b) HDX-MS exchange profile of 14-3-3ζ-strep and 14-3-3ζ in the ERα/14-3-3ζ protein complex after 20 seconds (blue), 2 minutes (green), 20 minutes (yellow) and 2 hours (red) of HDX. Plots show the amount of deuteration of each residue in the 14-3-3ζ protein. (c) HDX-MS exchange profile of 14-3-3ζ-strep (blue) and 14-3-3ζ in the ERα/14-3-3ζ protein complex (orange) after 20 seconds, 2 minutes, 20 minutes and 2 hours. Plots show the effect of ERα binding on each 14-3-3ζ residue at various time points. (d) HDX difference profile of ERα/14-3-3ζ complex minus 14-3-3ζ-strep after 2 hours incubation, which displays the effect of ERα binding on deuteration of 14-3-3ζ. Shielding effect (less deuterium exchange) is shown in blue and deshielding effects (increase in deuterium exchange) in red. Results are displayed on the 14-3-3ζ crystal structure. PDB: 6F09.

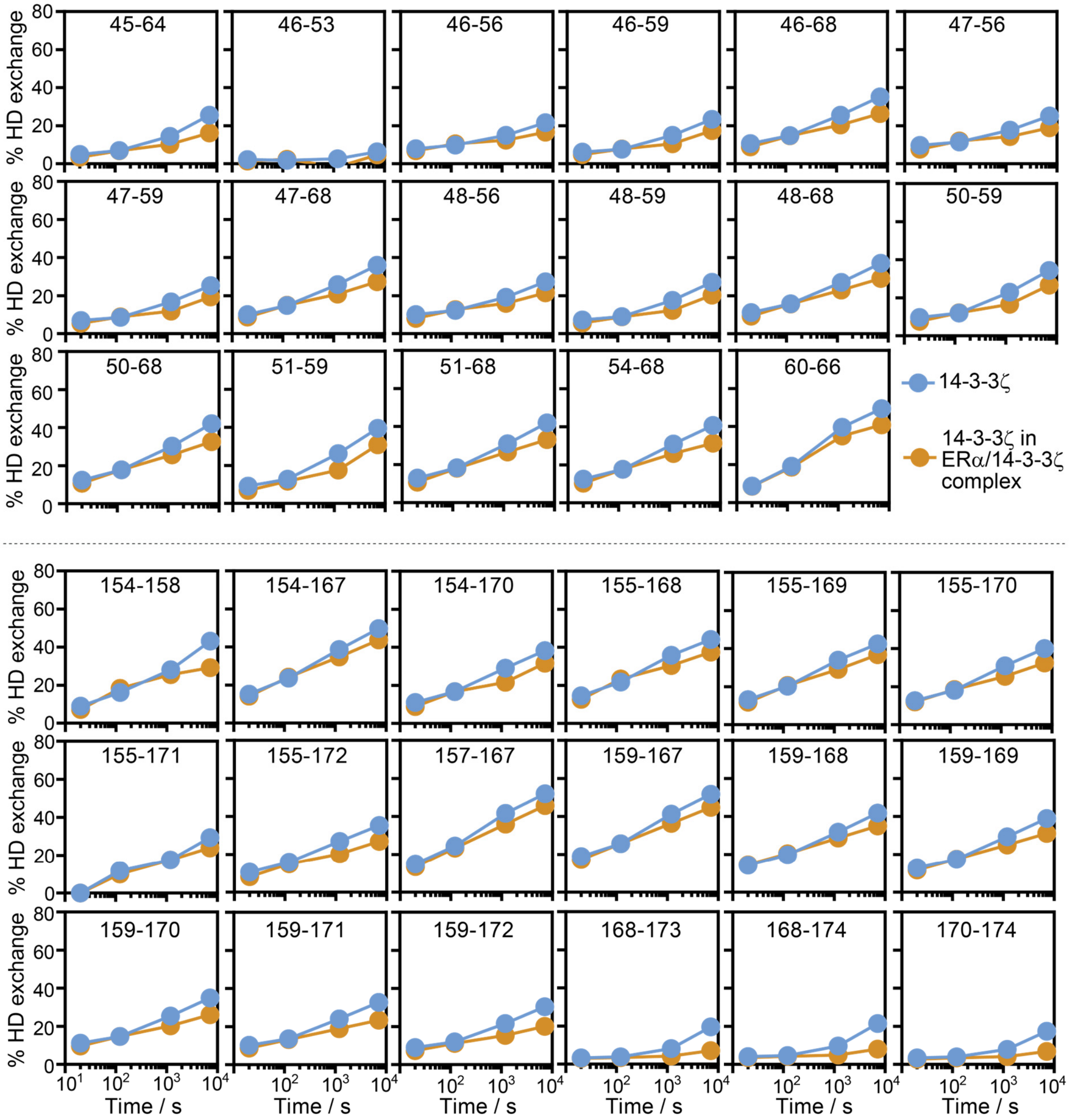

Figure S20a | HDX peptides of 14-3-3ζ. Hydrogen-deuterium exchange levels of peptide sequences within 14-3-3ζ after 20 seconds, 2 minutes, 20 minutes and 2 hours. Data is shown for 14-3-3ζ by itself (blue) and 14-3-3ζ within the ERα/14-3-3ζ complex (orange). Peptides are represented for regions that showed significant changes in HDX profiles after 2h incubation. Here shown, residues 45-66 in helix 3, and residues 154-174 in helix 6-7.

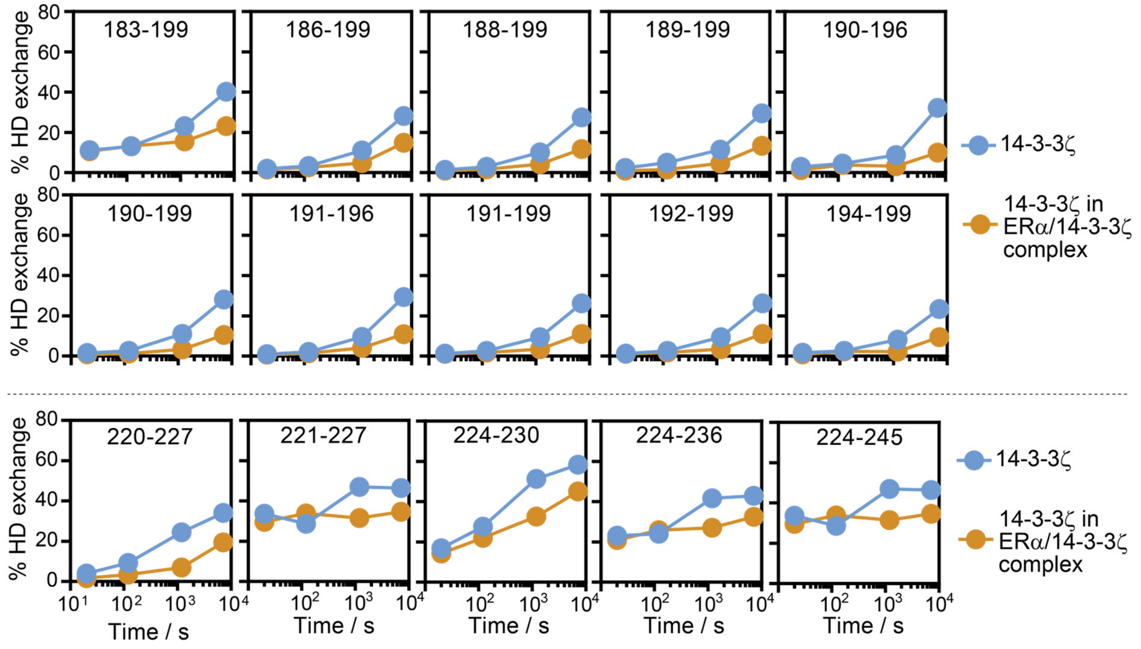

Figure S20b | HDX peptides of 14-3-3ζ. Hydrogen-deuterium exchange levels of peptide sequences within 14-3-3ζ after 20 seconds, 2 minutes, 20 minutes and 2 hours. Data is shown for 14-3-3ζ by itself (blue) and 14-3-3ζ within the ERα/14-3-3ζ complex (orange). Peptides are represented for regions that showed significant changes in HDX profiles after 2h incubation. Here shown, residues 183-199 in helix 8, and residues 220-245 in helix 9.

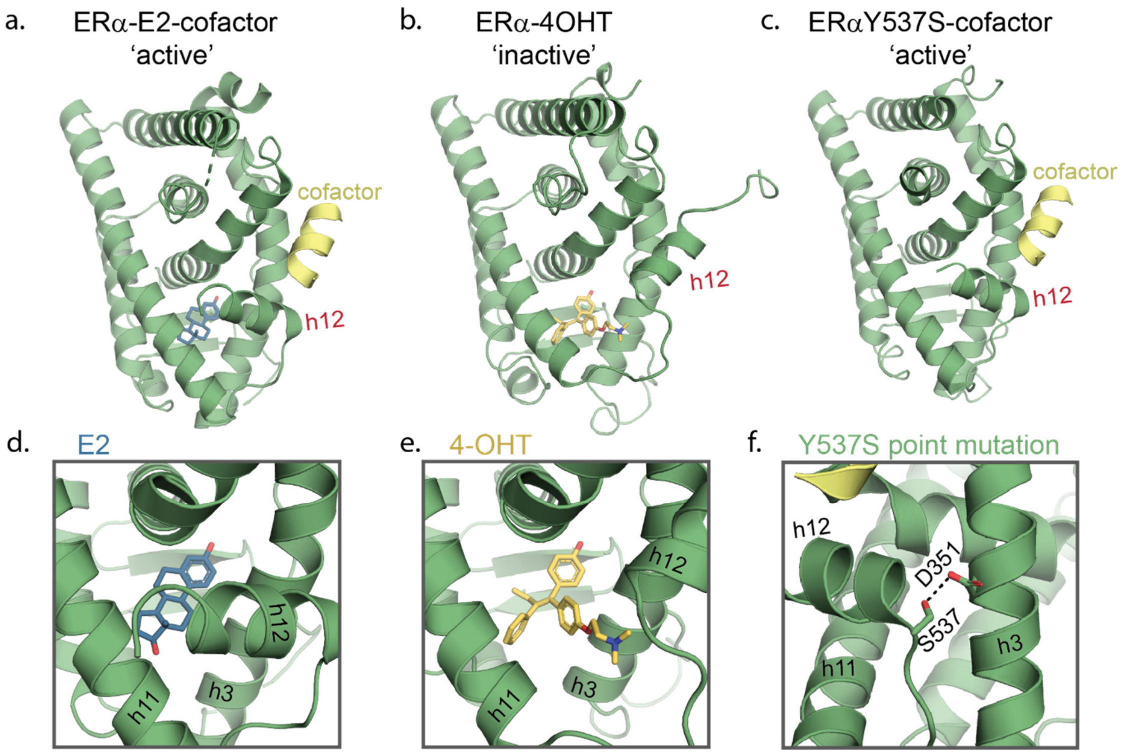

Figure S21 | ERα LDB structures. (a) Crystal structure of ERα ligand binding domain (LBD) (green cartoon) bound by E2 (blue sticks). Protein folds helix 12 in the active conformation allowing cofactor (yellow cartoon) binding. PDB: 5WGD (b) Crystal structure of ERα ligand binding domain (LBD) (green cartoon) bound by 4-OHT (yellow sticks). 4-OHT binding prevents helix 12 to fold in the proper conformation for cofactor binding. PDB: 3ERT (c) Crystal structure of ERα ligand binding domain (LBD) with point mutation Y537S (green cartoon). The Y537S mutants leads to protein folding of helix 12 in the active conformation, in absence of an agonistic ligand, allowing cofactor (yellow cartoon) binding. PDB: 2B23 (d-e) Zoomed image of E2 or 4-OHT binding within the ERα LBD, determining the fold of helix 12. (f) Point mutation Y537S at the N-terminal side of helix 12 and its interaction with D351 which stabilizes helix 12 to fold in an active conformation.

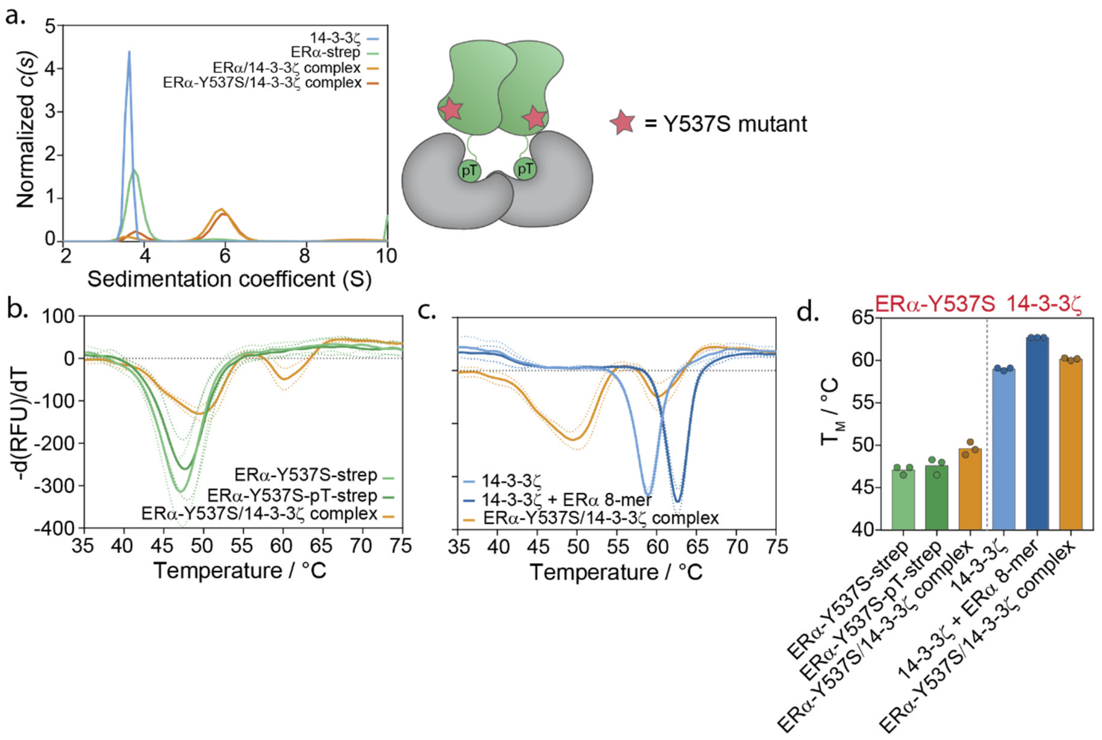

Figure S22 | SV-AUC and DSF 14-3-3ζ/ERα-Y537S mutant complex. (a) Area-normalized *c(s)* distributions of 10 μM 14-3-3ζ (blue), 10 μM ERα-strep (green), 10 μM ERα/14-3-3ζ complex (orange) and 10 μM ERα-Y537S/14-3-3ζ complex (dark orange). (b) Differential melting curve of 5 μM ERα-Y537S-strep (green), 5 μM ERα-Y537S-pT594-strep (green) and 10 μM ERα-Y537S/14-3-3ζ complex (orange). (c) Differential melting curve of 5 μM 14-3-3ζ (blue), 5 μM 14-3-3ζ + 50 μM ERα 8-mer phosphopeptide (blue) and 10 μM ERα-Y537S/14-3-3ζ complex (orange). (d) Melting temperatures (T_M) of ERα-Y537S and 14-3-3ζ individually and when present in complex. For data of wildtype ERα see SI Figure S16.

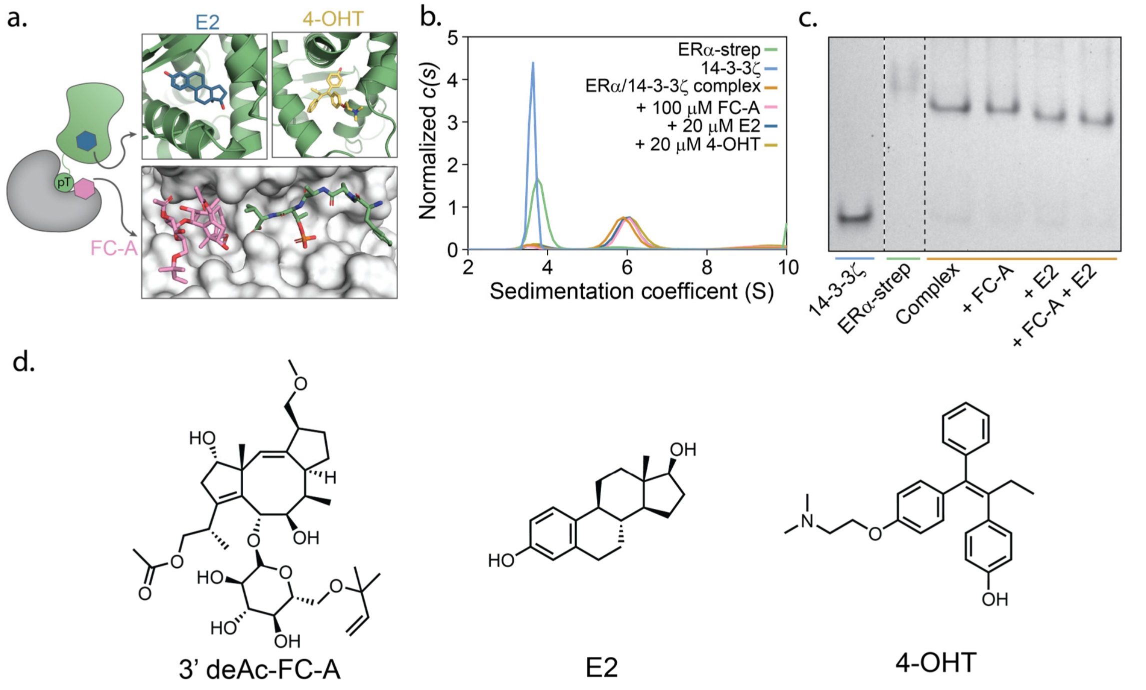

Figure S23 | Effect ligands on protein complex size. (a) Schematic representation of 14-3-3/ERα complex with ERα ligands (blue hexagon) binding in the ERα LBD or a PPI stabilizer (pink hexagon) binding at the 14-3-3/ERa protein interface. Crystal structures are given of ERα ligands E2 and 4-OHT in the ERα LDB (green cartoon) or FC-A binding at the 14-3-3σ (white surface)/ ERα (green sticks) interface. PDB: 5WGD & 3ERT. (b) Area-normalized c(s) distributions of 10 μM 14-3-3ζ (blue), 10 μM ERα-strep (green) and 10 μM ERα/14-3-3ζ complex (orange). The latter was also analyzed in presences, 100 μM 3'deAc-FC-A, 20 μM E2 (blue) or 20 μM 4-OHT (yellow). (c) Native page analysis of 2.5 μM 14-3-3ζ (blue), 2.5 μM ERα-strep (green) and the 5 μM ERα/14-3-3ζ complex. The latter is also analyzed in presence of 100 μM FC-A, 100 μM E2 or both. (d) Chemical structures of 3'deAc-FC-A, E2 and 4-OHT.

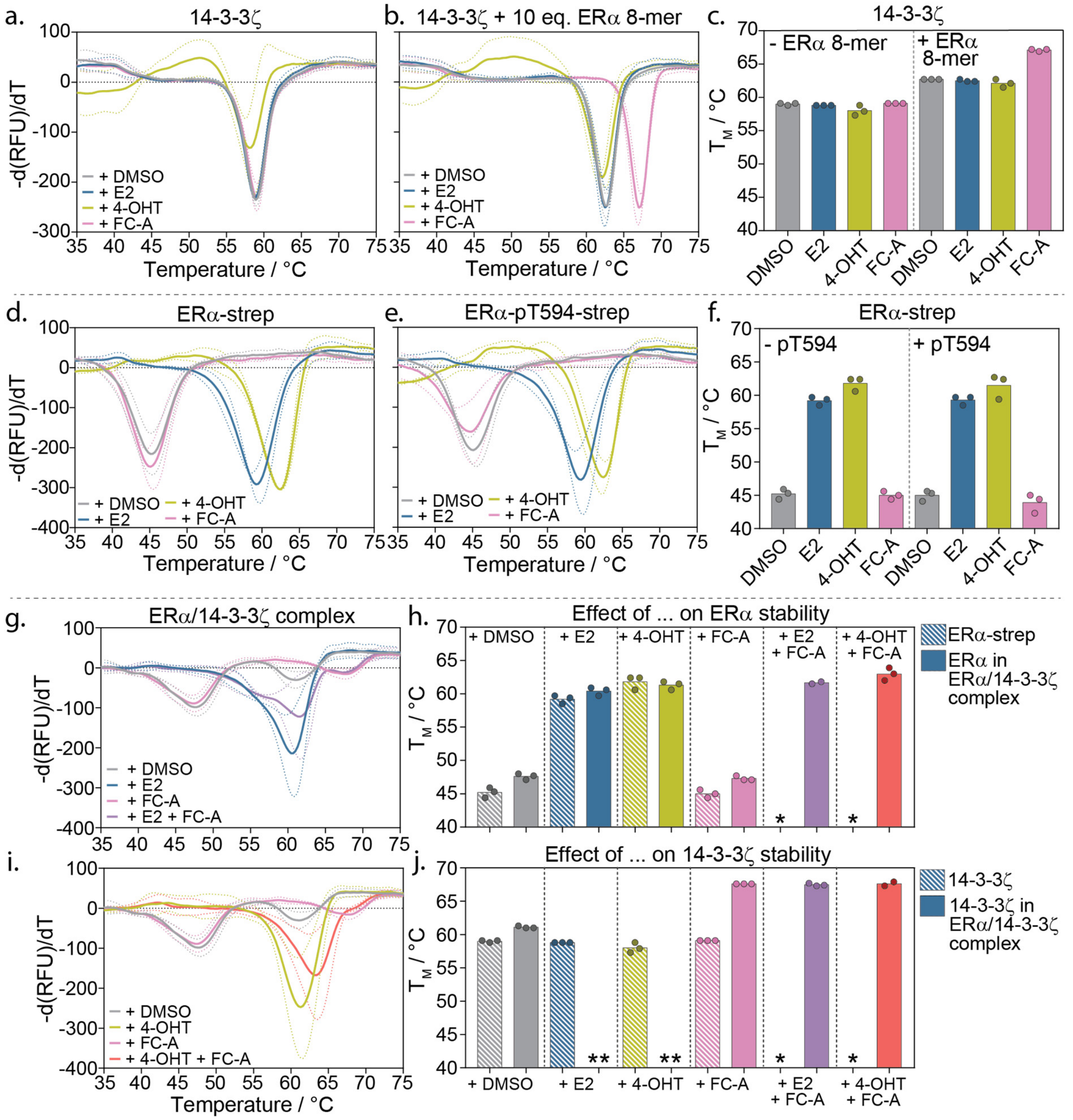

Figure S24 | DSF – ligand binding to 14-3-3ζ/ERα interaction. (a-b) Differential melting curves of 5 μM 14-3-3ζ-strep or 5 μM 14-3-3ζ + 50 μM ERα 8-mer phosphopeptide in presence of DMSO (negative control), 100 μM E2, 100 μM 4-OHT or 100 μM 3'deAc-FC-A. (c) Bar plot of melting temperatures of 5 μM 14-3-3ζ (+ 50 μM ERα 8-mer phosphopeptide) in presence of DMSO, (negative control), 100 μM E2, 100 μM 4-OHT or 100 μM 3'deAc-FC-A. (d-e) Differential melting curves of 5 μM ERα-strep or 5 μM ERα-pT594-strep in presence of DMSO (negative control), 100 μM E2, 100 μM 4-OHT or 100 μM 3'deAc-FC-A. (f) Bar plot of melting temperatures of (phosphorylated) ERα-strep in presence of DMSO, (negative control), 100 μM E2, 100 μM 4-OHT or 100 μM 3'deAc-FC-A. (g/i) Differential melting curves of 10 μM ERα/14-3-3ζ protein complex in presence of DMSO (negative control), 100 μM E2, 100 μM 4-OHT, 100 μM 3'deAc-FC-A and when combining 3'deAc-FC-A with either E2 or 4-OHT. (h) Bar plot representation of melting temperatures of ERα-strep and ERα in ERα/14-3-3ζ protein complex. It shows the effect of ligand binding on the thermal stability of ERa. (j) Bar plot representation of melting temperatures of 14-3-3ζ-strep and 14-3-3ζ in ERα/14-3-3ζ protein complex. It shows the effect of ligand binding on the thermal stability of ERα. * = not measured; ** = could not be determined from data.

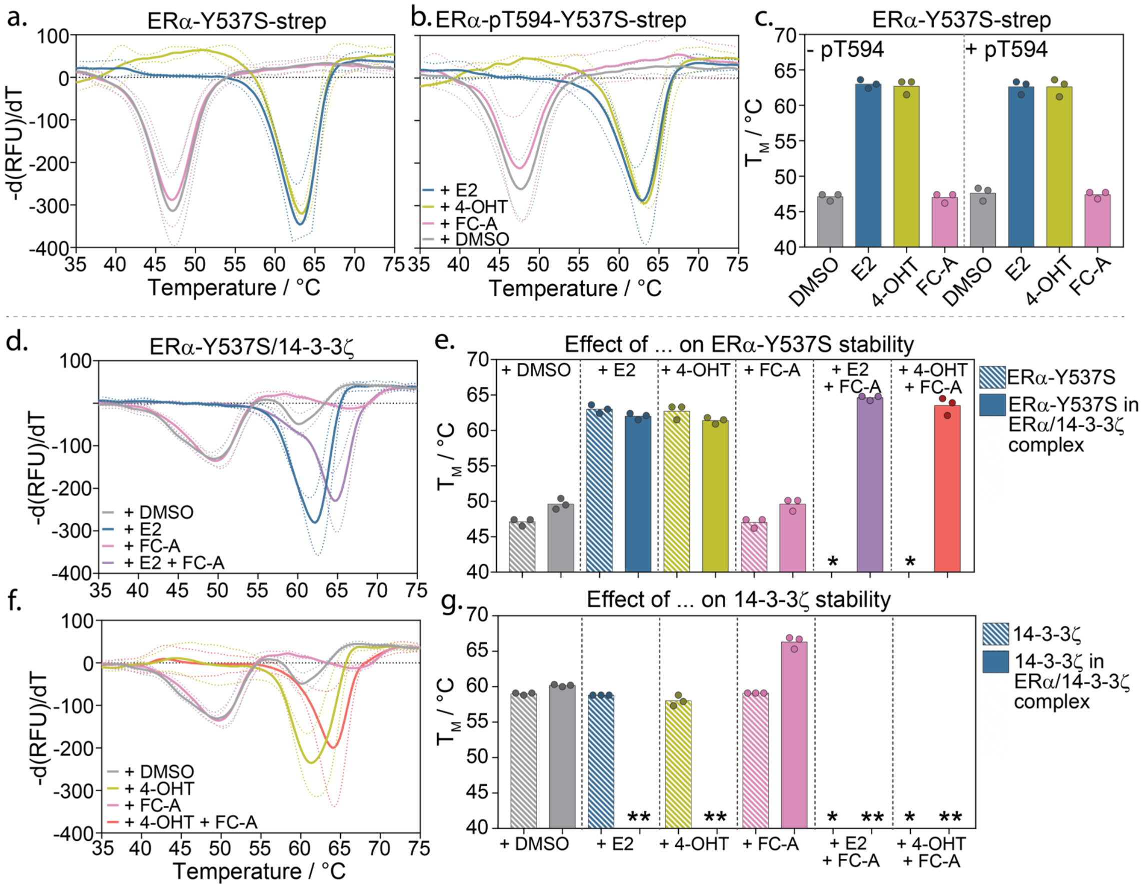

Figure S25 | DSF – ligand binding to 14-3-3ζ/ERα-Y537S interaction. (a-b) Differential melting curves of 5 μM ERα-Y537S-strep or 5 μM ERα-Y537S-pT594-strep in presence of DMSO (negative control), 100 μM E2, 100 μM 4-OHT or 100 μM 3'deAc-FC-A. (c) Bar plot of melting temperatures of (phosphorylated) ERα-Y537S-strep in presence of DMSO, (negative control), 100 μM E2, 100 μM 4-OHT or 100 μM 3'deAc-FC-A. (d/f) Differential melting curves of 10 μM ERα-Y537S/14-3-3ζ protein complex in presence of DMSO (negative control), 100 μM E2, 100 μM 4-OHT, 100 μM 3'deAc-FC-A and when combining 3'deAc-FC-A with either E2 or 4-OHT. (e) Bar plot representation of melting temperatures of ERα-Y537S-strep and ERα-Y537S in ERα-Y537S/14-3-3ζ protein complex. It shows the effect of ligand binding on the thermal stability of ERa. (g) Bar plot representation of melting temperatures of 14-3-3ζ-strep and 14-3-3ζ in ERα-Y537S/14-3-3ζ protein complex. It shows the effect of ligand binding on the thermal stability of ERα. * = not measured; ** = could not be determined from data. See data of wildtype ERa in SI Figure S24.

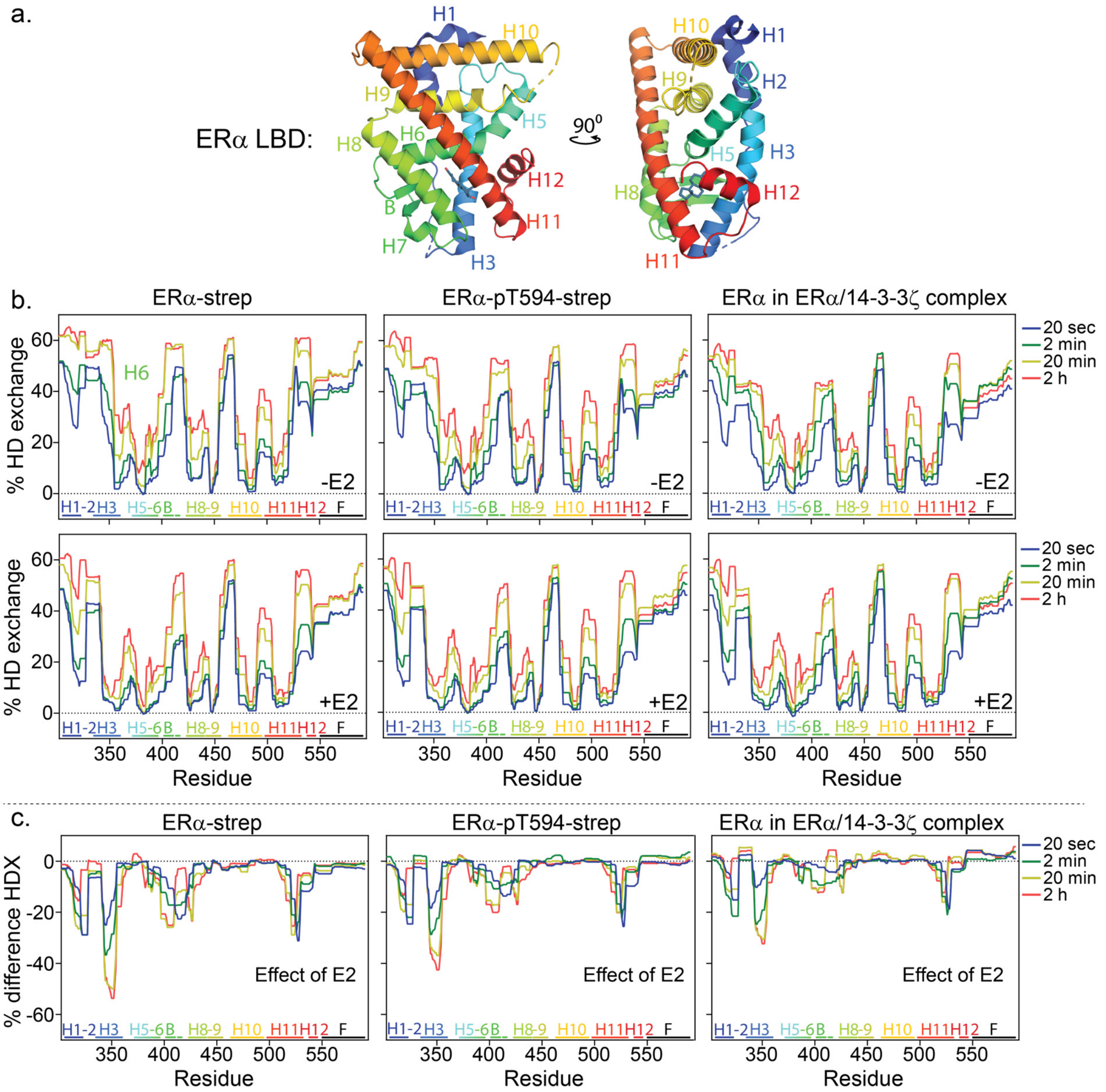

Figure S26 | HDX of E2 effect on ERα. (a) Crystal structure of ERα ligand binding domain (LBD) in rainbow colors with the N-terminal side colored in blue and the C-terminal side in red. Furthermore each alpha helix (H) and the beta sheets (B) are labelled with corresponding colors. PDB: 5WGD (b) HDX-MS exchange profile of ERα-strep, ERα-pT594-strep and ERα in the ERα/14-3-3ζ protein complex in absence (top) and presence (bottom) of E2 ligand after 20 seconds (blue), 2 minutes (green), 20 minutes (yellow) and 2 hours (red) of HDX. (c) Difference HDX exchange profile of ERα-strep, ERα-pT594-strep and ERα in ERα/14-3-3ζ protein complex upon E2 binding after 20 seconds, 2minutes, 20 minutes or 2 hours of HDX. Negative values implicate less HD exchange upon addition of E2 ligand whereas positive values implicate higher HD exchange upon addition of E2 ligand.

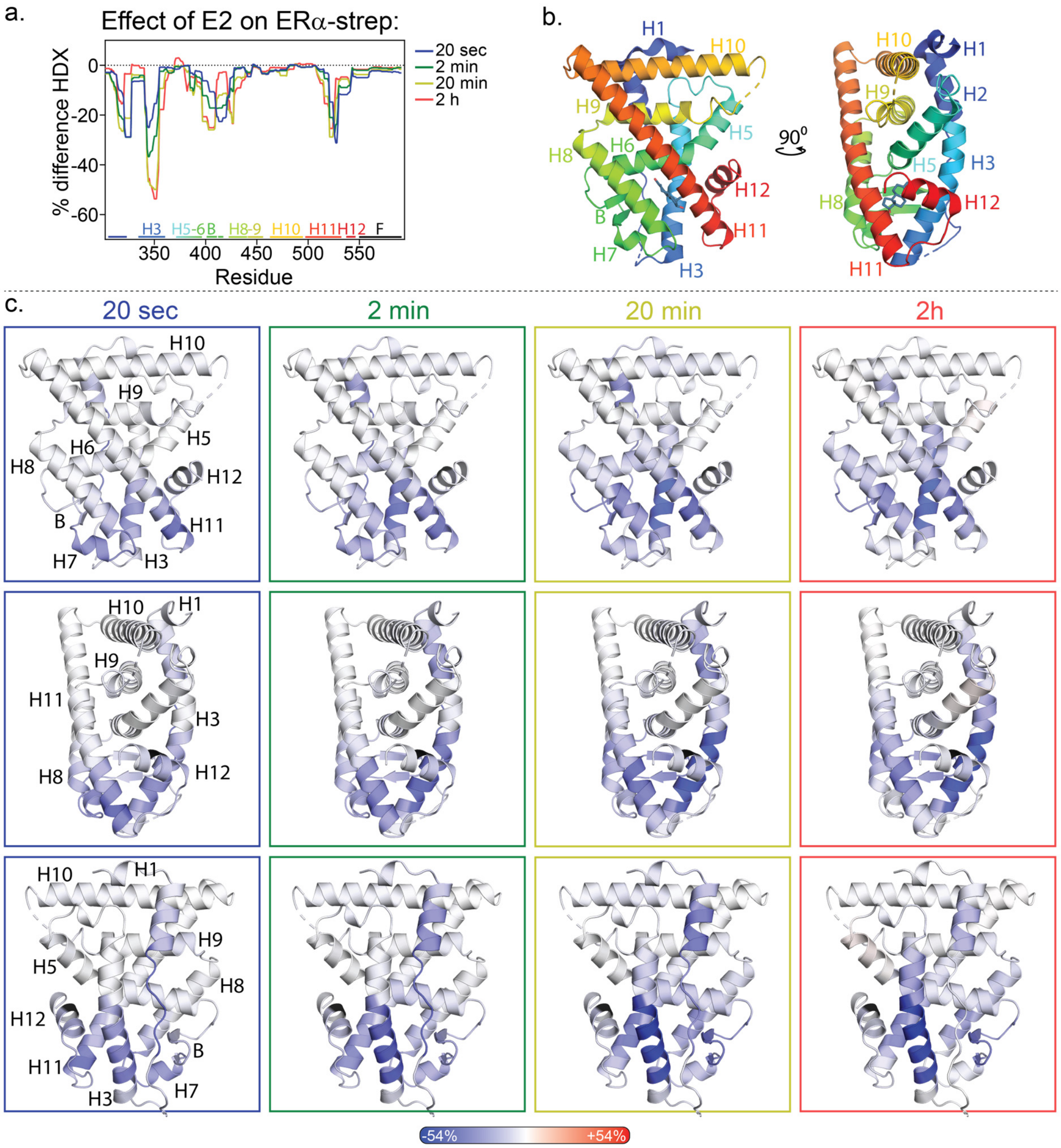

Figure S27 | HDX of E2 effect on ERα-strep. (a) Difference HDX exchange profile of ERα-strep upon E2 binding after 20 seconds, 2minutes, 20 minutes or 2 hours of HDX. Negative values implicate less HD exchange upon addition of E2 ligand whereas positive values implicate higher HD exchange upon addition of E2 ligand. (b) Crystal structure of ERα ligand binding domain (LBD) in rainbow colors with the N-terminal side colored in blue and the C-terminal side in red. Furthermore each alpha helix (H) and the beta sheets (B) are labelled with corresponding colors. PDB: 5WGD. (c) Difference HDX exchange profile of ERα-strep upon E2 binding after 20 seconds, 2minutes, 20 minutes or 2 hours of HDX, which displays the effect of E2 binding on deuteration of ERα. Shielding effect (less deuterium exchange) is shown in blue and deshielding effects (increase in deuterium exchange in red). Results are displayed on the ERα LBD crystal structure from three different sides. PDB: 5WGD.

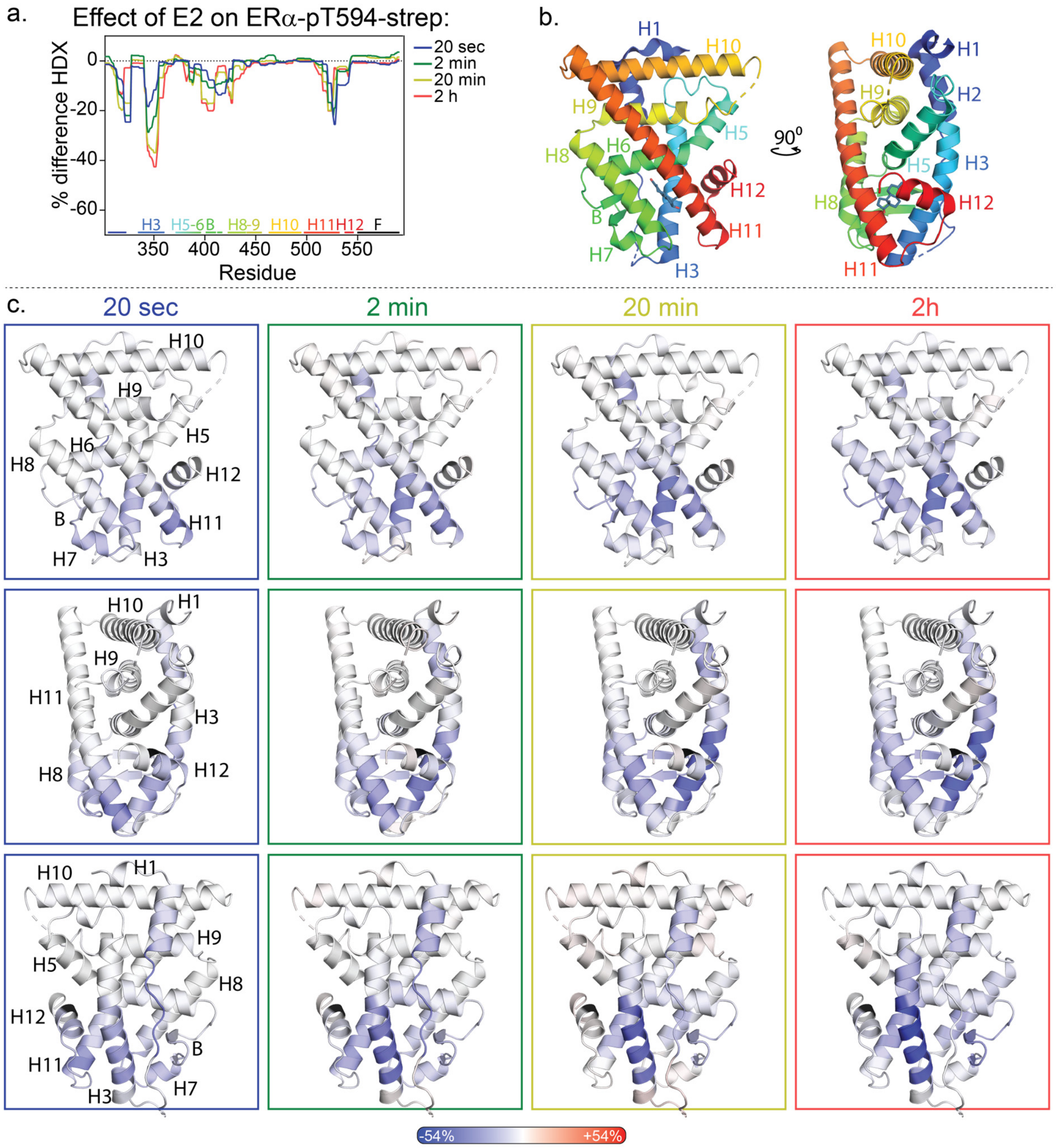

Figure S28 | HDX of E2 effect on ERα-pT594-strep. (a) Difference HDX exchange profile of ERα-pT594-strep upon E2 binding after 20 seconds, 2minutes, 20 minutes or 2 hours of HDX. Negative values implicate less HD exchange upon addition of E2 ligand whereas positive values implicate higher HD exchange upon addition of E2 ligand. (b) Crystal structure of ERα ligand binding domain (LBD) in rainbow colors with the N-terminal side colored in blue and the C-terminal side in red. Furthermore each alpha helix (H) and the beta sheets (B) are labelled with corresponding colors. PDB: 5WGD. (c) Difference HDX exchange profile of ERα-pT594-strep upon E2 binding after 20 seconds, 2minutes, 20 minutes or 2 hours of HDX, which displays the effect of E2 binding on deuteration of ERα. Shielding effect (less deuterium exchange) is shown in blue and deshielding effects (increase in deuterium exchange in red). Results are displayed on the ERα LBD crystal structure from three different sides. PDB: 5WGD.

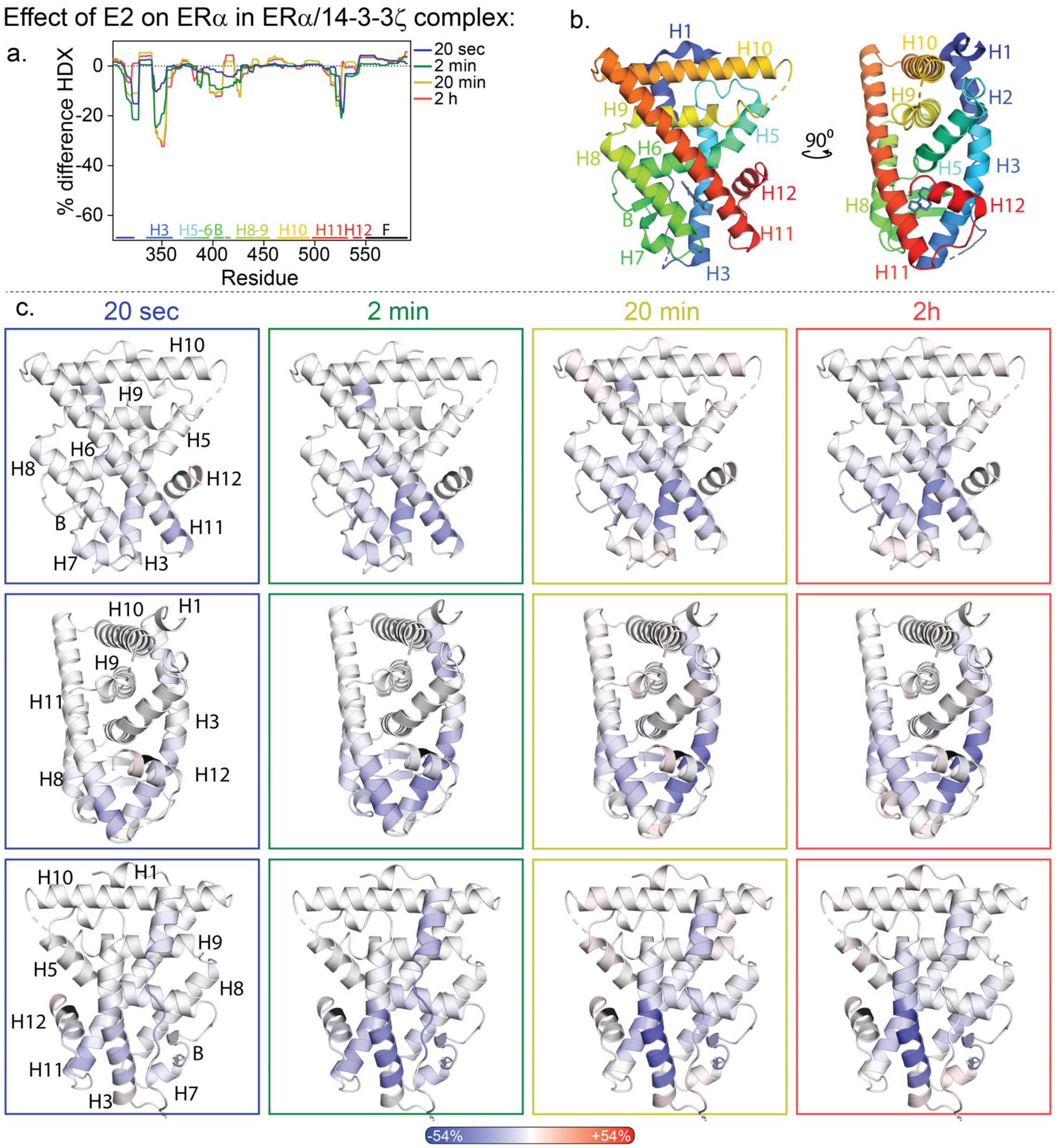

Figure S29 | HDX of E2 effect on ERα in ERα/14-3-3ζ protein complex. (a) Difference HDX exchange profile of ERα in ERα/14-3-3ζ protein complex upon E2 binding after 20 seconds, 2 minutes, 20 minutes or 2 hours of HDX. Negative values implicate less HD exchange upon addition of E2 ligand whereas positive values implicate higher HD exchange upon addition of E2 ligand. (b) Crystal structure of ERα ligand binding domain (LBD) in rainbow colors with the N-terminal side colored in blue and the C-terminal side in red. Furthermore each alpha helix (H) and the beta sheets (B) are labelled with corresponding colors. PDB: 5WGD. (c) Difference HDX exchange profile of ERα in ERα/14-3-3ζ upon E2 binding after 20 seconds, 2minutes, 20 minutes or 2 hours of HDX, which displays the effect of E2 binding on deuteration of ERα. Shielding effect (less deuterium exchange) is shown in blue and deshielding effects (increase in deuterium exchange in red). Results are displayed on the ERα LBD crystal structure from three different sides. PDB: 5WGD.

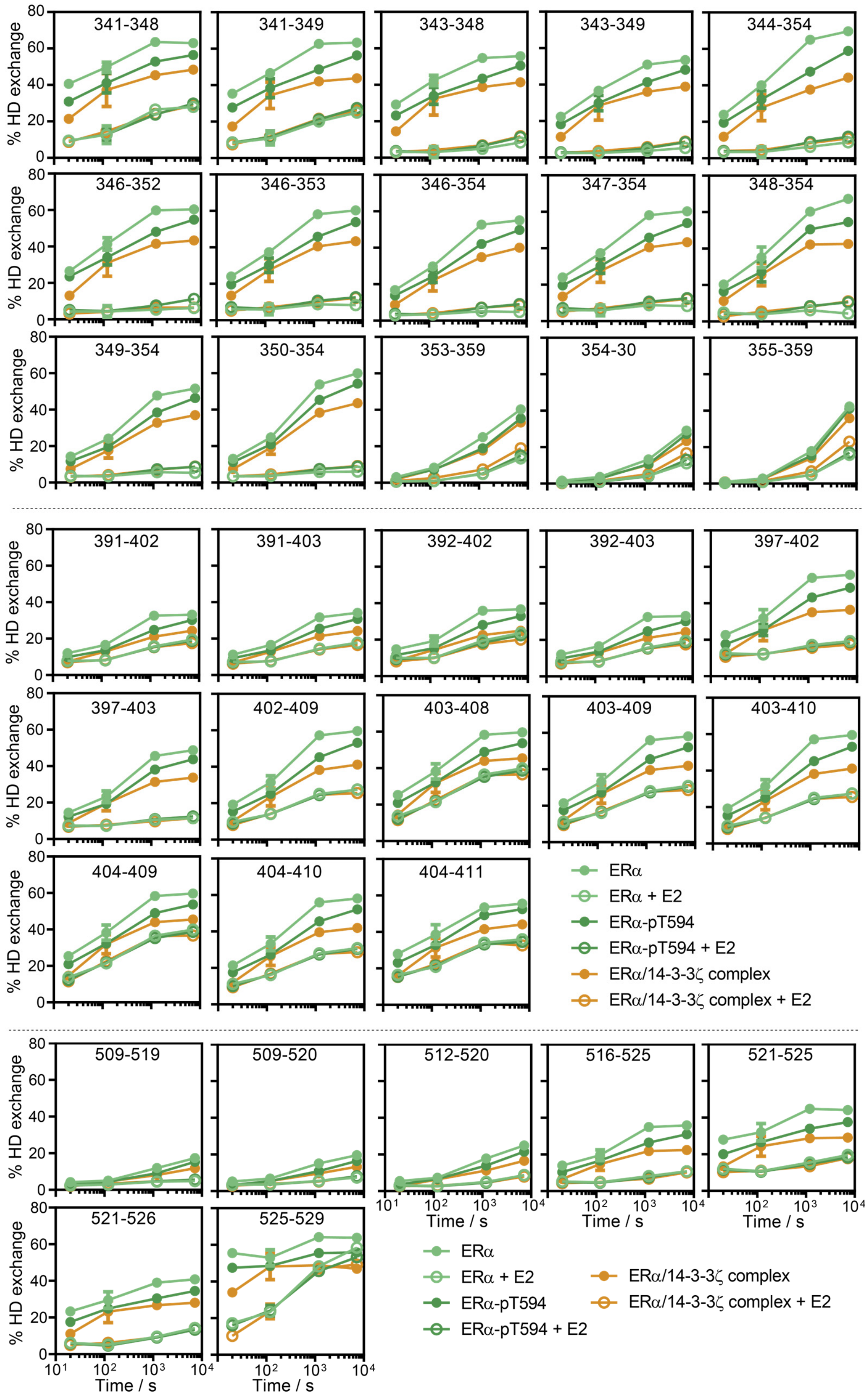

Figure S30 | HDX peptides of ERα upon E2 binding. Hydrogen-deuterium exchange levels of peptide sequences within ERα, ERα-pT594 and ERα-pT594 in the ERα/14-3-3ζ protein complex after 20 seconds, 2 minutes, 20 minutes and 2 hours. Data is shown for ERα (light green) ERα-pT594 (dark green) and ERα-pT594 in the ERα/14-3-3ζ protein complex (orange) in presence and absence of E2 (open circles). Peptides are represented for regions that showed significant changes in HDX profiles after 2h incubation. Here shown, residues 341-361 in helix 3, residues 398-410 in beta sheets and helix 7, and residues 512-527 in helix 11.

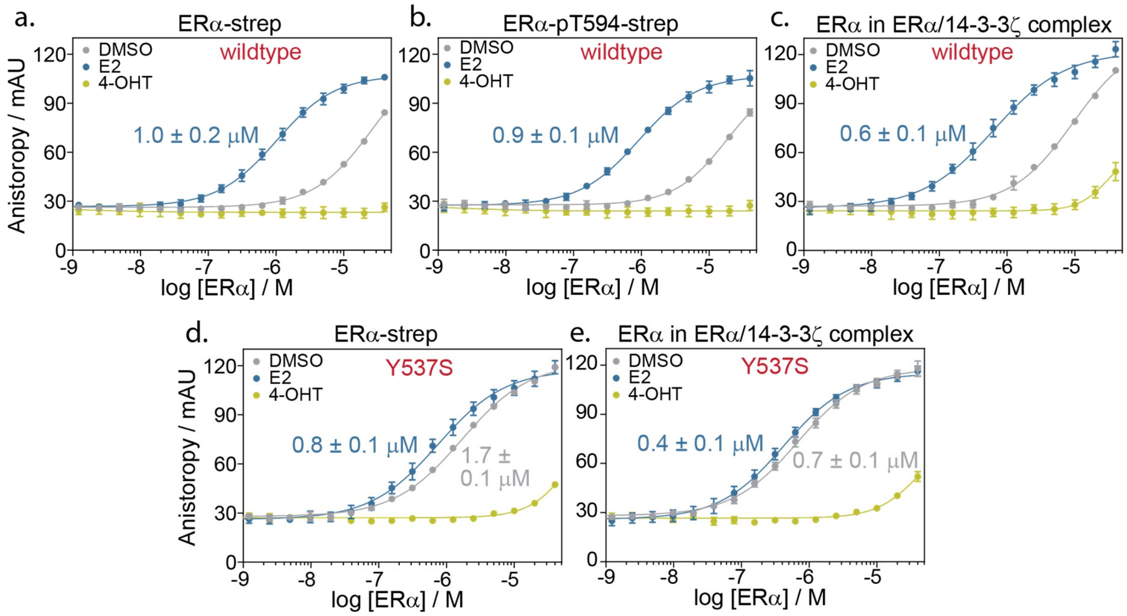

Figure S31 | Effect PKA mutations on 14-3-3 binding. Fluorescence anisotropy assay of various ERα proteins titration to 10 nM fluorescein-labelled SRC-1 cofactor peptide in presence of DMSO (negative control, grey), 100 μM E2 (blue) or 100 μM 4-OHT (yellow). Results shown are an average and standard deviation of three independent measurements.

Table S1. Data collection and refinement statistics of crystal structures

**PDB**	**8C40**	**8C42**	**8C3Z**	**8C43**
Protein	14-3-3σΔc	14-3-3σΔc	14-3-3σΔc	14-3-3σΔc
Peptide	ERα(PKA)-pT594	ERα(PKA)-pT594	ERα(PKA)-pT594-strep	ERα(PKA)-pT594-strep
Ligand	-	3'deAc-FC-A	-	3'deAc-FC-A
Beam	DESY p11	DESY p11	DESY p11	DESY p11
Data collection				
Wavelength (Å)	1.0332	1.0332	1.0332	1.0332
Space group	C 2 2 21	C 2 2 21	C 2 2 21	C 2 2 21
Cell dimensions<br>a, b, c (Å)<br>α, β, γ (°)	 82.1 112.0 62.8<br>90, 90, 90	 82.7 111.8 62.6<br>90, 90, 90	 82.2 112.4 62.4<br>90, 90, 90	 81.8 112.0 62.3<br>90, 90, 90
Resolution (Å)	45.57 – 1.40 (1.42 – 1.40)	45.41 – 1.40 (1.43 – 1.40)	45.47 – 1.40 (1.43 – 1.40)	45.34 – 1.40 (1.42 – 1.40)
I / σ(I)	24.9 (4.5)	19.4 (2.4)	24.3 (2.5)	23.8 (2.7)
Completeness (%)	99.6 (97.2)	97.2 (92.1)	99.9 (98.9)	95.5 (88.2)
Redundancy	13.3 (12.6)	13.6 (13.1)	13.3 (12.2)	13.9 (13.4)
$CC_{1/2}$	0.999 (0.930)	0.999 (0.837)	1.000 (0.798)	1.000 (0.780)
Refinement				
No. reflections	56873	54755	57050	53888
R_{work}/R_{free}	0.165/0.184	0.180/0.196	0.174/0.186	0.181/0.193
No. atoms<br>Protein<br>Ligand/ion<br>Water	 1990<br>4<br>322	 1943<br>48<br>267	 1992<br>4<br>307	 1999<br>48<br>249
B-factors<br>Protein<br>Ligand/ion<br>Water	 14.91<br>18.59<br>26.12	 18.33<br>20.79<br>28.48	 17.09<br>21.18<br>28.08	 18.87<br>26.90<br>28.84
R.m.s. deviations<br>Bond lengths (Å)<br>Bond angles (°)	 0.006<br>0.91	 0.345<br>4.25	 0.006<br>0.89	 0.346<br>4.10
Ramachandran<br>favored (%)<br>outliers (%)	 98.73<br>0.00	 98.30<br>0.00	 98.233<br>0.00	 97.86<br>0.00

Protein constructs

14-3-3ζ-strep **pETDuet-1**

MDKNELVQKAKLAEQAERYDDMAACMKSVTEQGAELSNEERNLLSVAYKNVVGARRSSWRVVSSIEQKTEGAEKK
QQMAREYREKIETELRDICNDVLSLLEKFLIPNASQAESKVFYLKMKGDYYRYLAEVAAGDDKKGIVDQSQQAYQ
EAFEISKKEMQPTHPIRLGLALNFSVFYYEILNSPEKACSLAKTAFDEAIAELDTLSEESYKDSTLIMQLLRDNL
TLWTSDTQGDEAEAGEGGENWSHPQFEK

His-SUMO-ERαLBD-F(PKA)-Strep **pCDFDuet-1**

MGHHHHHHGGSDSEVNQEAKPEVKPEVKPETHINLKVSDGSSEIFFKIKKTTPLRRLMEAFAKRQGKEMDSLRFL
YDGIRIQADQTPEDLDMEDNDIIEAHREQIGGKKNALALSLTADQMVSALLDAEPPILYSEYDPTRPFSEASMMG
LLTNLADRELVHMINWAKRVPGFVDLTLHDQVHLLECAWLEILMIGLVWRSMEHPGKLLFAPNLLLDRNQGKCVE
GMVEIFDMLLATSSRFRMMNLQGEEFVCLKSIILLNSGVYTFLSSTLKSLEEKDHIHRVLDKITDTLIHLMAKAG
LTLQQQHQRLAQLLLILSHIRHMSNKGMEHLYSMKCKNVVPLYDLLLEMLDAHRLHAPTSRGGASVEETDQSHLA
TAGSTSSHSLQKYYITG**EAEGRRATV**PWSHPQFEK

His-SUMO-ERαLBD-F(PKA) **pCDFDuet-1**

MGHHHHHHGGSDSEVNQEAKPEVKPEVKPETHINLKVSDGSSEIFFKIKKTTPLRRLMEAFAKRQGKEMDSLRFL
YDGIRIQADQTPEDLDMEDNDIIEAHREQIGGKKNALALSLTADQMVSALLDAEPPILYSEYDPTRPFSEASMMG
LLTNLADRELVHMINWAKRVPGFVDLTLHDQVHLLECAWLEILMIGLVWRSMEHPGKLLFAPNLLLDRNQGKCVE
GMVEIFDMLLATSSRFRMMNLQGEEFVCLKSIILLNSGVYTFLSSTLKSLEEKDHIHRVLDKITDTLIHLMAKAG
LTLQQQHQRLAQLLLILSHIRHMSNKGMEHLYSMKCKNVVPLYDLLLEMLDAHRLHAPTSRGGASVEETDQSHLA
TAGSTSSHSLQKYYITG**EAEGRRATV**

SUMO-PKA **pACYC**

MSDSEVNQEAKPEVKPEVKPETHINLKVSDGSSEIFFKIKKTTPLRRLMEAFAKRQGKEMDSLRFLYDGIRIQAD
QTPEDLDMEDNDIIEAHREQIGGMGNAAAAKKGSEQESVKEFLAKAKEDFLKKWETPSQNTAQLDQFDRIKTLGT
GSFGRVMLVKHKESGNHYAMKILDKQKVVKLKQIEHTLNEKRILQAVNFPFLVKLEFSFKDNSNLYMVMEYVAGG
EMFSHLRRIGRFSEPHARFYAAQIVLTFEYLHSLDLIYRDLKPENLLIDQQGYIQVTDFGFAKRVKGRTWTLCGT
PEYLAPEIILSKGYNKAVDWWALGVLIYEMAAGYPPFFADQPIQIYEKIVSGKVRFPSHFSSDLKDLLRNLLQVD
LTKRFGNLKNGVNDIKNHKWFATTDWIAIYQRKVEAPFIPKFKGPGDTSNFDDYEEEEIRVSINEKCGKEFTEF

ABC = His tag
ABC = SUMO tag
ABC = 14-3-3 binding sequence
ABC = mutated amino acids (S305A, F591R, P592R)
ABC = strep-tag
